# Triplin, a small molecule, reveals copper ion transport in ethylene signaling from ATX1 to RAN1

**DOI:** 10.1371/journal.pgen.1006703

**Published:** 2017-04-07

**Authors:** Wenbo Li, Randy F. Lacey, Yajin Ye, Juan Lu, Kuo-Chen Yeh, Youli Xiao, Laigeng Li, Chi-Kuang Wen, Brad M. Binder, Yang Zhao

**Affiliations:** 1 Institute of Plant Physiology and Ecology, Shanghai Institutes for Biological Sciences, Chinese Academy of Sciences, Shanghai, China; 2 University of Chinese Academy of Sciences, Shanghai, China; 3 Department of Biochemistry and Cellular and Molecular Biology, University of Tennessee, Knoxville, Tennessee, United States of America; 4 Agricultural Biotechnology Research Center, Academia Sinica, Taipei, Taiwan; 5 National Key Laboratory of Plant Molecular Genetics, CAS Center for Excellence in Molecular Plant Sciences, Shanghai, China; 6 Faculty of Life Science and Technology, Kunming University of Science and Technology, 68 Wenchang Road, Yunnan, China; The University of North Carolina at Chapel Hill, UNITED STATES

## Abstract

Copper ions play an important role in ethylene receptor biogenesis and proper function. The copper transporter RESPONSIVE-TO-ANTAGONIST1 (RAN1) is essential for copper ion transport in *Arabidopsis thaliana*. However it is still unclear how copper ions are delivered to RAN1 and how copper ions affect ethylene receptors. There is not a specific copper chelator which could be used to explore these questions. Here, by chemical genetics, we identified a novel small molecule, triplin, which could cause a triple response phenotype on dark-grown *Arabidopsis* seedlings through ethylene signaling pathway. *ran1-1* and *ran1-2* are hypersensitive to triplin. Adding copper ions in growth medium could partially restore the phenotype on plant caused by triplin. Mass spectrometry analysis showed that triplin could bind copper ion. Compared to the known chelators, triplin acts more specifically to copper ion and it suppresses the toxic effects of excess copper ions on plant root growth. We further showed that mutants of ANTIOXIDANT PROTEIN1 (ATX1) are hypersensitive to tiplin, but with less sensitivity comparing with the ones of *ran1-1* and *ran1-2*. Our study provided genetic evidence for the first time that, copper ions necessary for ethylene receptor biogenesis and signaling are transported from ATX1 to RAN1. Considering that triplin could chelate copper ions in *Arabidopsis*, and copper ions are essential for plant and animal, we believe that, triplin not only could be useful for studying copper ion transport of plants, but also could be useful for copper metabolism study in animal and human.

## Author summary

Copper ions are cofactors of protein functions, and their disorder is closely related to many human diseases which drive to develop new copper chelator related drugs. In plants, copper ions are essential for ethylene receptors, but many details are unclear. Researchers need novel specific copper chelators to study these questions. Here, by using plant chemical genetics, we identified a novel chemical triplin, which could activate ethylene signaling pathway by chelating copper ions essential for ethylene receptors. Ethylene resistance mutant *etr1-1* and *ein2* were resistant to triplin, and copper transporter mutants *ran1-1* and *ran1-2* were hypersensitive to triplin. Using triplin as a molecular genetics tool, we showed copper chaperone ATX1 acts the upstream of RAN1 which transports copper ions to ethylene receptors. We provide a sample that metabolism could be studied by combining chemical genetics and known signaling pathway. Moreover, triplin could chelate copper ions effectively and specifically in *Arabidopsis*, and could be a useful tool to study copper ion transport in plant, and valuable for designing copper chelator related drug.

## Introduction

The phytohormone ethylene (C_2_H_4_) plays important roles in plant growth and development. When exposed to ethylene gas for 3 days, dark-grown *Arabidopsis* seedling shows a typical triple response phenotype including a short hypocotyl and root, larger diameter hypocotyl, and an exaggerated apical hook [1,2]. ETHYLENE RESPONSE1 (ETR1), ETHYLENE RESPONSE2 (ETR2), ETHYLENE RESPONSE SENSOR1 (ERS1), ETHYLENE RESPONSE SENSOR2 (ERS2) and ETHYLENE INSENSITIVE4 (EIN4) are five ethylene receptors in *Arabidopsis* which function redundantly and negatively to regulate ethylene signaling and responses. The receptors signal is transferred to a downstream Raf-like protein kinase CONSTITUTIVE TRIPLE RESPONSE1 (CTR1) [3,4]. CTR1 interacts with and phosphorylates an endoplasmic reticulum (ER) membrane-localized Nramp homolog ETHYLENE IN SENSITIVE 2 (EIN2). This prevents EIN2 from activating downstream components of ethylene signaling including ETHYLENE INSENSITIVE3 (EIN3) and EIN3-LIKE1 (EIL1). When ethylene binds to the receptors, the phosphorylation of EIN2 by CTR1 is reduced, leading to accumulation of EIN2 and proteolytic cleavage of the cytosolic C-terminal domain of EIN2, which enters the nucleus to initiate ethylene signaling [5–10].

Copper ions are cofactors that are required for ethylene binding to ETR1. Ethylene insensitive mutant *etr1-1* eliminates both ethylene binding and the interaction of copper ion with the receptor [11]. In *Arabidopsis*, the copper ions required by the ethylene receptors are transported by RAN1, also called HEAVY METAL ATPASE 7 (HMA7), which is a Cu-transporting P-type ATPase. Two weak mutant alleles, *ran1-1* and *ran1-2*, were identified in a mutant screening where they responded to the ethylene receptor antagonist trans-cyclooctene (TCO) with a triple response phenotype which could be partially suppressed by adding copper ion to the plant growth medium. Additionally, *ran1-1* and *ran1-2* are hypersensitive and display a similar phenotype upon treatment with a low concentration of the copper ion chelator neocuproine. It has been shown that RAN1 is essential for the biogenesis of ethylene receptors in *Arabidopsis* [12–14].

A similar protein, Ccc2a, has been identified in *Saccharomyces cerevisiae* where it functions to transport copper ions from the copper chaperone, Atx1, to the secretory pathway. Atx1 is a small metal homeostasis factor that protects cells against reactive oxygen toxicity caused by excess or abnormal distribution of copper ions [15–18]. A homolog of Atx1 is found in humans. This metallochaperone, HUMAN ATX1-LIKE HOMOLOG1 (HAH1), also transports copper ions and interacts with Menkes and the Wilson disease proteins [19–21].

In *Arabidopsis*, there are two homologs of yeast Atx1, COPPER CHAPERONE (CCH) and ATX1 [22–24]. A T-DNA insertion mutant of ATX1 is specifically hypersensitive to excess copper ion as well as copper deficiency, while the over-expression of *ATX1* enhances plant tolerance to excess copper ion and copper deficiency [25]. Previous yeast two-hybrid assays suggested that ATX1 and CCH△ (without C-terminal) interact with RAN1 and HMA5 [24,26]. It has been predicted that copper ions transported by RAN1 that are essential for ethylene receptors may come from ATX1 and CCH, but there is no genetic evidence to support this hypothesis [25,27].

To date, only the chelator neocuproine has been found to cause a plant triple response phenotype, but whether the phenotype could be suppressed by adding copper ions is unknown [14]. Neocuproine should be cautiously used as it may potentiate a cytotoxic effect of endogenous copper on cells [28]. Thus, there is no report about a specific copper ion chelator which causes a triple response phenotype specifically via a reduction in copper ions. We believe that a copper ion chelator with such a property would be useful for investigating the mechanisms of copper ion transport to the ethylene receptors. More ever, chemical genetics approaches are powerful in dealing with the function redundancy of components involved in phytohormone signaling, and have helped scientists to identify ABA receptors and novel signaling components involving in auxin and other phytohormones [29–32].

Here, we have used a chemical genetics approach to uncover a novel synthetic small molecule triplin. We found that triplin could cause a triple response phenotype in dark-grown *Arabidopsis* seedlings as a copper chelator. By testing the sensitivity of copper ion transport mutants to triplin, we showed that ATX1 acts upstream of RAN1. Our genetic and biochemical results support a model where ATX1 transports copper ions to RAN1 for ethylene receptor biogenesis and signaling. Triplin does not directly act on ethylene binding to receptor. Rather, it perturbs copper ion transport involved in the interaction of RAN1 and ATX1. In addition, triplin suppresses the toxic effects of excess copper ion on plant root growth. Thus, triplin may provide a useful chemical genetics tool to study copper ion transport in plants, and could be a lead structure for drug development for human copper disorders diseases in the future.

## Results

### Identification and characterization of triplin

**Fig 1:**
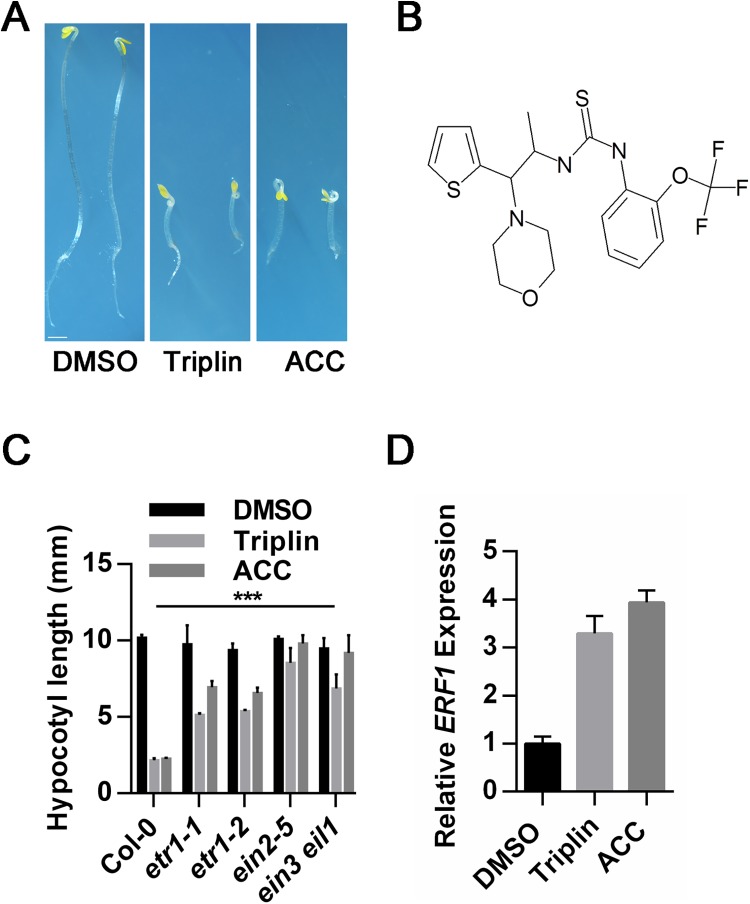
Triplin acts through ethylene signaling pathway to cause a triple response phenotype in *Arabidopsis* seedlings. (A) Phenotypes of 3-day-old, dark-grown Col-0 seedlings treated with 100 μM triplin, 50 μM ACC, or 1% (v/v) DMSO as a control. Scale bar represents 1 mm. (B) The chemical structure of triplin. (C) The hypocotyl length of 3-day-old, dark-grown Col-0, *etr1-1*, *etr1-2*, *ein2-5 and ein3 eil1* seedlings treated with 100 μM triplin, 50 μM ACC, or 1% (v/v) DMSO as a control. Each experiment was repeated three times, more than 30 seedlings were used every time. Error bars represent SEM. ***P < 0.0001 (two-tailed Student’s t-test) indicated a significant difference of the hypocotyl length of the mutants compared to Col-0 treated with 100 μM triplin. (D) qRT-PCR analysis the expression of the ethylene response gene *ERF1* treated by 1% (v/v) DMSO, 100 μM triplin, or 50 μM ACC. Each experiment was repeated three times, and error bars represent SEM.

To identify novel small molecules that affect ethylene signaling, we used the same plant chemical genetics screening method described previously [32–34]. *Arabidopsis* wild type Columbia-0 (Col-0) were grown and screened against a synthetic small molecule library with 12,000 compounds in the dark for 3 days. Those compounds that caused a triple response in seedlings were selected and their chemical genetics effects were retested. From this plant chemical screening, we acquired 14 compounds which cause a triple response in dark-grown *Arabidopsis* seedlings. Among these hits, the 5 strongest hits representing different structures were further assayed. Using the ethylene perception inhibitor, AgNO_3_, and the ethylene insensitive mutant *ein2-5*, we found that only a compound we called triplin (1-(1-morpholino-1-(thiophen-2-yl) propan-2-yl)-3-(2-(trifluoromethoxy) phenyl) thiourea) worked through the ethylene signaling pathway (Fig 1 and S1 Fig). We also examined the chemical genetic activities of 38 triplin analogs collected from the library. Our results showed 5 analogs could cause triple response phenotypes at 100 μM, and 11 analogs caused the phenotype at a higher concentration of 200 μM. The structure analysis of these active analogs indicates that the morpholino and thiourea moieties of the molecules could be important for enhancing their chemical genetics activities (S1 Table).

### Triplin acts through the ethylene signal transduction pathway

Two strategies were employed to study the mechanism of action of triplin. First, we screened ethyl methanesulfonate (EMS) mutagenized populations of Col-0 for triplin resistant mutants. We acquired nine triplin resistant mutants which also showed resistance to the application of the ethylene biosynthesis precursor, 1-aminocylopropane-1-carboxylic acid (ACC). Among these resistant mutants, two are dominant mutants that have the same mutation as *etr1-1* as revealed by DNA sequencing [1]. The other seven mutants are recessive and were genetically determined to be on the *EIN2* locus by examining *F1*s acquired from crossings with *ein2* (S2 Fig). We further assayed the effects of triplin treatment on known ethylene signaling mutants. Ethylene resistant mutant *etr1-1*, *etr1-2*, *ein2-5* and *ein3 eil1* showed resistance to triplin (Fig 1C and S3 Fig). Additionally, adding 500 μM AgNO_3_ blocked triplin’s effects on seedlings (S6C Fig). Consistent with triplin affecting ethylene signaling, the ethylene-responsive gene, *ERF1*, was up-regulated by triplin treatment (Fig 1D). These results indicated that triplin acts on ethylene signaling. It is possible that triplin also increases ethylene biosynthesis to cause a triple response. To determine if this was occurring, we first examined how alterations in ethylene biosynthesis affect responses to triplin. We found that triplin does not increase *ACS*s gene expression levels. Neither the ACC synthase (ACS) inhibitor, aminoethoxyvinylglycine (AVG) nor the ethylene biosynthesis mutant *cin5* affects triplin responses. Additionally, triplin treatment did not enhance ethylene biosynthesis and actually causes a decrease in ethylene production (S4 Fig). Together, these results indicate that triplin treatments cause triple responses by acting on ethylene signaling but not ethylene biosynthesis.

### Triplin genetically acts upstream of ethylene receptors in the ethylene signaling network

**Fig 2:**
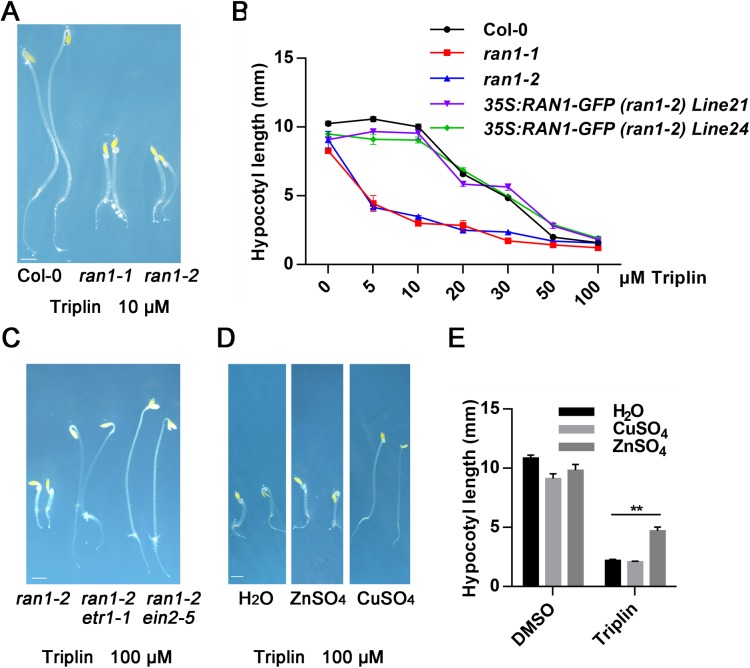
*ran1-1* and *ran1-2* are hypersensitive to triplin and copper can partially reverse the effects of triplin. (A) Phenotypes of 3-day-old, dark-grown seedlings of Col-0, *ran1-1* and *ran1-2* treated with 10 μM triplin. (B) Triplin dose responses of Col-0, *ran1-1*, *ran1-2* and two *35S*:*RAN1-GFP (ran1-2)* transgenic lines. Data is the average hypocotyl length under each condition. Each experiment was repeated three times, more than 30 seedlings were used every time. Error bars represent SEM. (C) Phenotypes of 3-day-old, dark-grown seedlings of *ran1-2*, *ran1-2 etr1-1* and *ran1-2 ein2-5* treated with 100 μM triplin. (D) The phenotypes of 3-day-old, dark-grown seedlings of Col-0 treated with 100 μM triplin in the presence of 20 μM ZnSO_4_, 20 μM CuSO_4_ or H_2_O as a control. (E) The hypocotyl length of seedlings as described in (D). Each experiment was repeated three times, more than 30 seedlings were used every time. Error bars represent SEM. **p < 0.01 indicated the difference of the hypocotyl length between the seedlings treated with CuSO_4_ compared with H_2_O and ZnSO_4_ in the presence of 100 μM triplin. Scale bars represent 1 mm.

To map where triplin acts on the ethylene signaling network, we assayed how triplin treatments affect other ethylene insensitive mutants of ethylene receptors including *ers1-1*, *ers2-1*, *etr2-1* and *ein4*. Our results showed they were all resistant to triplin (S3B Fig), indicating that triplin likely acts on or upstream of the ethylene receptors in the ethylene signaling network. Previous research has shown that the copper ion transporter RAN1 is involved in the biogenesis of ethylene receptors and certain ion chelators such as neocuproine can trigger a plant triple response [14]. As we expected, *ran1-1* and *ran1-2* mutants were hypersensitive to triplin treatments comparable with the published results for neocuproine [14] (S10B and S10C Fig). Low doses of triplin (e.g. less than 10 μM) had no visible effects on wild type but caused a triple response in *ran1-1* and *ran1-2* (Fig 2A). Further, introducing RAN1 into the mutants rescued a wild type response to triplin (Fig 2B). However, *ran1-2 etr1-1* and *ran1-2 ein2-5* double mutants were both resistant to triplin treatments similar to the *etr1-1* and *ein2-5* single mutants (Fig 2C). Together, our chemical genetics results showed that triplin is likely to act upstream of the ethylene receptors and may be involved in altering copper ion transport to the receptors. Our simple postulation is that triplin is a copper ion chelator which causes the triple response phenotype by chelating the copper ions necessary for ethylene receptor biogenesis.

### Triplin chelates copper ions

**Fig 3:**
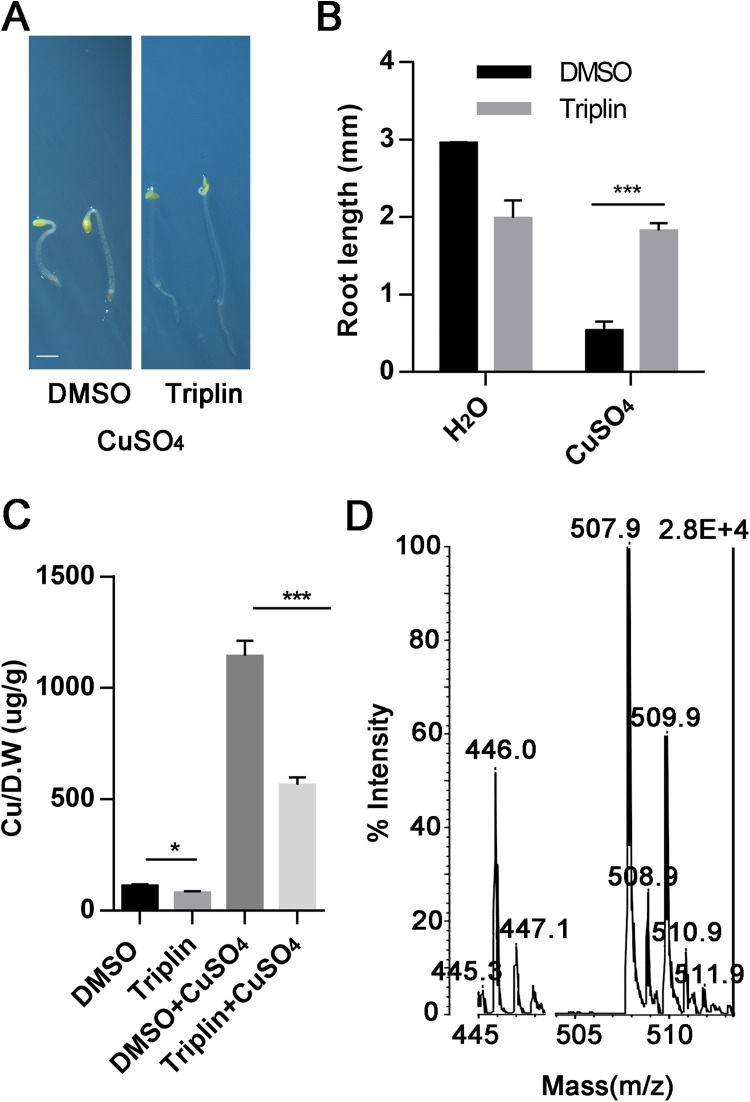
Triplin can chelate copper ions *in vitro*. (A) The phenotypes of 3-day-old, dark-grown seedlings of Col-0 treated with 50 μM CuSO_4_ in the presence of 1% (v/v) DMSO or 100 μM triplin. Scale bar represents 1 mm. (B) The root length of the seedlings as described in (A). Each experiment was repeated three times, more than 30 seedlings were used every time. Error bars represent SEM. (C) The relative copper contents of 3-day-old, dark-grown Col-0 seedlings on 0.5xMS growth medium with or without 100 μM triplin and/or 20 μM CuSO_4_. D.W represents dry weight of the seedlings. Each experiment was repeated three times (Error bars represent SEM). (D) MALDI-TOF-MS analysis of the mixture of equal volume of 100 μM CuSO_4_ and 100 μM triplin. Mr of triplin is 446.0g/mol. Mr of triplin+Cu is 510.0g/mol. *P < 0.05 and ***P < 0.0001 (two-tailed Student’s t-test) indicate a significant difference between groups of different treatments.

To determine if triplin acts as a copper ion chelator and its specificity, we examined the effects of copper ions and other ions such as Ag, Zn, Ca, Mg, Mn, Co, Ni, Na, K, Li and Mo on triplin responses in plants. Our results showed that addition of excess Cu^2+^ (CuSO_4_) partially reverses the effects of triplin on plants (Fig 2D and 2E). Of the other ions tested, only Ag^+^ (AgNO_3_) had a similar effect (S5 Fig and S6C Fig). We also examined the known copper ion chelator neocuproine and found that its effects were reversed by adding Zn^2+^ (ZnSO_4_) under our assay conditions (S10A Fig). To further verify that triplin acts by chelating copper ions, we tested whether triplin treatment can alleviate the toxic effects of high levels of copper ions on plant growth. Our results indicated that the growth of *Arabidopsis* seedlings roots treated with 50 μM CuSO_4_ was strongly inhibited; addition of 100 μM triplin at the same time partially reversed this inhibition of root growth (Fig 3A and 3B). Higher concentration of CuSO_4_ causes more severe growth inhibition which can also be partially reversed by adding triplin (S6A and S6B Fig). We next tested if neocuproine has a similar effect as triplin on plant grown with high levels of copper ions. To our surprise, neocuproine aggravated the copper ion toxic effects on plant growth. Another thing is that ZnSO_4_ could restore the phenotype caused by neocuproine. This indicated that the specificity of neocuproine is questionable (S10A and S10C Fig).

To better understand the effects of triplin on plants, we measured the relative copper ion content of seedlings grown with copper ions and triplin. When seedlings were grown on a normal 0.5xMS growth medium, adding 100 μM triplin to the medium slightly decreased the copper ion content in the seedlings from 110 μg/g to 80 μg/g. When seedlings were grown on a growth medium containing 20 μM CuSO_4_, the relative copper ion content in the seedlings increased from 110 μg/g to 1,100 μg/g. Adding 100 μM triplin reduced the ion content from 1,100 μg/g to 560 μg/g (Fig 3C). We tried to mix triplin with different metal ions such as Ag, Cu, Zn and Ca. Unexpectedly, we found that triplin formed a tawny turbidness with CuSO_4_, and a black precipitate with AgNO_3_ (S7 Fig). We analyzed the products formed from mixing 100 μM CuSO_4_ with 100 μM triplin by Matrix-Assisted Laser Desorption/ Ionization Time of Flight Mass Spectrometry (MALDI-TOF-MS) analysis. With this we observed a peak in the m/z of 509.9, which is equal to the sum of molecular weights of triplin (446.0 g/mol) and copper (Fig 3D). Silver ions can also conjugate with triplin, but a higher concentration (e.g. 10 mM) of AgNO_3_ is needed. Conjugates of triplin with other metal ions including ZnSO_4_ and FeSO_4_ were not detected with our mass spectrographic analysis (S8 Fig). Together, our results indicate that triplin is a copper chelator which can also chelate silver to a less degree. This possibly explains why both Cu^2+^ and Ag^+^ can block or partially block triplin’s effects on plant growth.

### Triplin causes the triple response phenotype by affecting copper ion transport in ethylene signaling

**Fig 4:**
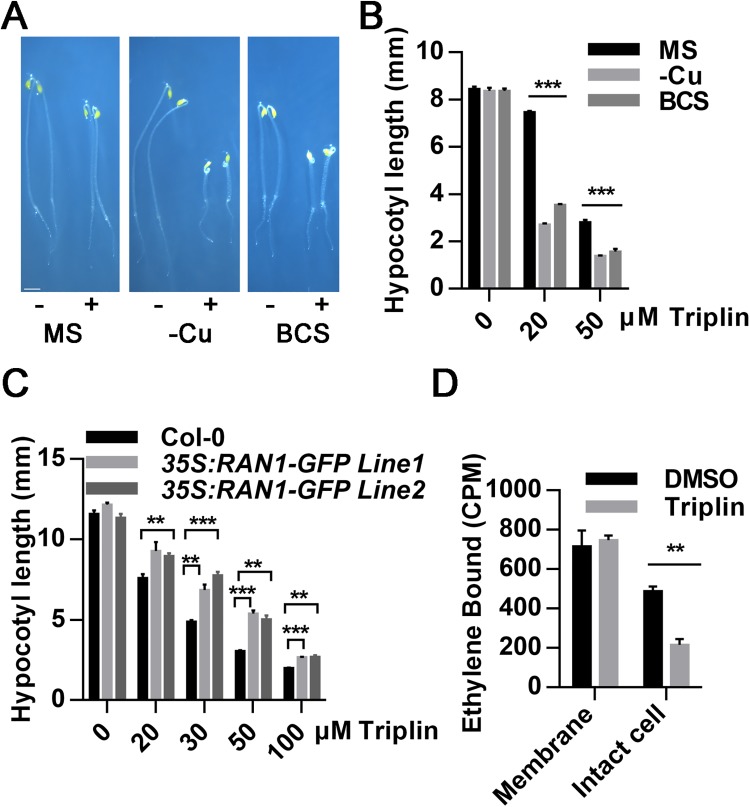
Triplin can affect the transport of copper ions *in vivo*. (A) The phenotypes of 3-day-old, dark-grown seedlings of Col-0 on different growth medium with (+) or without (-) 20 μM triplin. -Cu represents the growth medium made of all essential elements needed for plant growth except copper ion. BCS represents the growth medium made of 0.5xMS salt with 500 μM of the copper ion chelator BCS. Scale bar represents 1 mm. (B) The hypocotyl length of 3-day-old seedlings as described in (A). The difference of the hypocotyl lengths represent either the seedlings grown on -Cu or BCS medium compared to the ones grown on 0.5xMS. (C) The hypocotyl length of 3-day-old seedlings of Col-0 and *35S*: *RAN1-GFP* lines grown in dark under different dose of triplin. (D) Triplin’s effects on ethylene-binding to ETR1 expressed in yeast. Saturable ethylene binding to intact yeast cells expressing the ethylene binding domain of ETR1 and membranes isolated from these yeast cells was measured. Ethylene binding is indicated as counts per minute (CPM). Each experiment was repeated three times, and error bars represent SEM. In (B) and (C), experiments were repeated three times, more than 30 seedlings were used every time. Error bars represent SEM. *P < 0.05, **P < 0.01, ***P < 0.0001 (two-tailed Student’s t-test) indicate a significant difference between groups of different treatments.

In order to examine how triplin acts as a copper ion chelator to perturb ethylene signaling and cause the triple response phenotype, we first used two copper ion deficient growth medium to grow plants. One included all essential elements for plant growth except copper ions were not added. The other consisted of 0.5xMS growth medium supplemented with 500 μM of the copper ion chelator, bathocuproinedisulfonic acid (BCS). The effects of 20 μM or 50 μM triplin on plant growth in dark were compared on these medium to control medium. The seedlings grown on both copper-deficient growth medium showed more severe triple responses in response to 20 μM triplin than seedlings grown on control medium (Fig 4A and 4B). Under these same growth conditions with different triplin concentration, *ein2-5* showed obvious resistance to triplin (S9 Fig). This indicates that the exaggerated triple response in the absence of added copper is also dependent on ethylene signaling.

These results are consistent with our earlier results indicating that triplin can chelate copper ions. However, we also noticed that simply lowering copper levels in the growth medium is not enough to cause a triple response. This led us to speculate that there may be additional actions of triplin on plants to cause the triple response. One possibility we considered was that triplin enters into plant cells to affect copper ion transport. To examine this possibility, we used *RAN1* overexpression transgenic lines in the Col-0 background to test their sensitivity to triplin treatment. Our results showed that the overexpression lines are resistant to triplin treatments (Fig 4C and S14 Fig). One possible explanation is that triplin acts as copper ion chelator that competes for copper with the ethylene receptors resulting in less ethylene binding to the receptors. To examine this possibility, we measured ethylene binding to membranes isolated from yeast expressing the ethylene binding domain of ETR1 in the presence and absence of triplin [14]. Triplin had no measurable effect on specific ethylene binding to the receptors (Fig 4D). By contrast, ethylene binding to the receptors was reduced approximately 50% when triplin was added to the growing yeast cells expressing ETR1 (Fig 4D). These results indicate that triplin is not directly affecting ethylene binding but may affect the delivery of copper ions to the receptors by affecting other copper delivery proteins. For example, triplin treatments may reduce the copper ion levels below the optimal levels needed for some copper ion chaperones. Lack of copper ions would result in a lower number of functional receptors leading to the triple response. If this is true, we should find some copper ion transport mutants that are either hypersensitive or resistant to triplin treatment.

### ATX1 acts on copper ion transport to ethylene receptors

**Fig 5:**
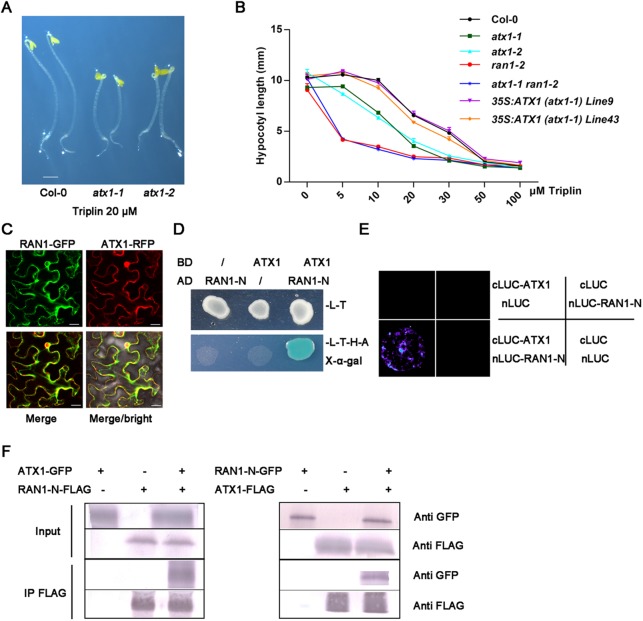
The copper chaperone ATX1 interacts with RAN1. (A) *atx1-1* and *atx1-2* are hypersensitive to triplin. The phenotypes of 3-day-old, dark-grown Col-0, *atx1-1* and *atx1-2* seedlings treated with 20 μM triplin are shown. Scale bar represents 1 mm. (B) Hypocotyl lengths of 3-day-old, dark-grown seedlings of Col-0, *atx1-1*, *atx1-2*, *ran1-2*, *atx1-1 ran1-2* and two *35S*:*ATX1-GFP (atx1-1)* transgenic lines treated with different doses of triplin are shown. Each experiment was repeated three times, more than 30 seedlings were used every time. Error bars represent SEM. (C) Subcellular co-localization of ATX1 and RAN1. ATX1-RFP and RAN1-GFP were transiently expressed in N. benthamiana leaves and observed and imaged under a confocal microscope. Scale bars represent 20 μM. (D) ATX1 interacted with RAN1 in yeast two-hybrid assay. ATX1 was fused to a GAL4 DNA-binding domain (BD) and RAN1-N (289 amino-terminal amino acids of RAN1) was ligated to a GAL4 activation domain (AD). The protein interactions were examined on cells grown on synthetic dropout (-Leu/-Trp/-His/-Ade) medium plus X-α-Gal (50mg/L) plates for 3 days. (E) Bimolecular fluorescence complementation assays showed interaction between ATX1 and RAN1 using the split luciferase system. Nicotiana benthamiana leaves were infiltrated with agrobacteria containing different construct combinations harboring both the C- and N-terminal of the luciferase fused to either ATX1 and RAN1-N or just one of them (controls). (F) Co-Immunoprecipitation assays showed interaction between ATX1 and RAN1. Nicotiana benthamiana leaves were infiltrated with agrobacteria containing ATX1-GFP/FLAG and RAN1-N-FLAG/GFP or just one of them (controls). The protein extracts were immunoblotted with anti-FLAG antibody or anti-GFP antibody.

Previous studies showed RAN1 is a key copper ion transporter involved in ethylene receptor biogenesis [12–14]. However the mechanisms for how copper ions are delivered to RAN1 and how RAN1 delivers copper to the ethylene receptors are presently missing. Therefore, we took advantage of triplin to look for the protein(s) that deliver copper to RAN1. We speculated that mutations in such proteins should be hypersensitive to triplin treatment. We therefore tested triplin sensitivity of T-DNA mutants of *Arabidopsis* that affect copper ion transport, including *copt1*, *copt2*, *copt4*, *copt5*, *hma1*, *hma5*, *hma6*, *ccs*, *zip2*, *zip4*, *cch* and *atx1*. This uncovered that only the copper ion chaperone mutants *atx1-1* and *atx1-2* are hypersensitive to triplin (Fig 5A and 5B). ATX1 is a copper ion chaperone playing an essential role in copper ion homeostasis that confers plant tolerance to both copper excess and deficiency conditions in *Arabidopsis* [25]. *atx1-1* (SALK_026221) is a knock-out mutant identified previously [25], *atx1-2* (SALK_041022) is another knock-out mutant identified in this research (S11 Fig). Both mutants showed hypersensitivity to triplin treatments. Application of 20 μM triplin to *atx1-1* and *atx1-2* seedlings caused a triple response as seen with the exaggerated apical hook and shorter hypocotyl. By contrast, 20 μM triplin did not cause a triple response phenotype in wild type seedlings. Re-introducing the *ATX1* gene into the *atx1-1* mutants restored a wild type response to triplin (Fig 5B). The hypersensitivity of *atx1-1* to triplin could be partially reduced by adding copper. Furthermore, *atx1-2 etr1-1* and *atx1-1 ein2-5* double mutants were resistant to triplin treatment comparable with the *etr1-1* and *ein2-5* single mutants (S11C Fig). This indicates that triplin is acting to result in lower levels of copper delivered to RAN1, which in turn, reduces deliver of copper to the ethylene receptors.

### ATX1 physically interacts with RAN1

Previous work showed that the ethylene receptors are localized at ER membrane network [35]. To further examine how ATX1 is involved in RAN1 mediated copper ion transport to ethylene receptors, we compared the subcellular localization of ATX1 and RAN1. First, we used 5-day-old light grown *35S*:*ATX1-GFP (atx1-1)* transgenic lines and observed filar and cloudy GFP signals in the cytoplasm and around the nucleus of the root meristem zone cells. In root elongation zone cells, stronger GFP signal were observed in the cytoplasm and nucleus. Follow up observations using 4', 6-diamidino-2-phenylindole (DAPI) stain confirmed the nuclear signals observed in *35S*:*ATX1-GFP (atx1-1)* transgenic lines [24, 25] (S12A and S12B Fig). For the transgenic lines grown in dark, we observed strong GFP signals in the cytoplasm and nucleus of the hypocotyl cells comparable with what was observed in root elongation zone cells (S12C Fig). We speculate that, in mature cells, the expression and signal of ATX1-GFP is stronger than in younger cells. We then carried out plasmolysis experiments and showed that these GFP signals observed in intact cells are not localized at the cell wall. We next scanned a thin layer of a cell and observed similar filar and cloudy GFP signals, which may be from ATX1-GFP adhered to the cell endomembrane system (S12D Fig). Using a transgenic line expressing both ATX1-GFP and WAK2-mCherry, an ER marker, we observed the ATX1-GFP signals were partially co-localized with the ER markers (S12E Fig).

To confirm this observation, we also used a transient expression system to express ATX1-GFP and WAK2-mCherry in tobacco (*Nicotiana benthamiana*) leaf epidermal cells to observe the sub-cellular location of ATX1. The observations in these experiments are consistent with our previous observations that ATX1 is localized to the cytoplasm and nucleus and may adhere to ER membranes (S13A Fig). Using this transient expression system, we observed that GmMAN1-mcherry (a Golgi marker) displayed a marked punctate signal and OsREM4.1-mCherry (a plasma membrane marker) showed no filar signals, which are different from the ATX1-GFP pattern. This confirmed that ATX1 did not specifically localize to the plasma membrane and Golgi. Only WAK2-mCherry showed filar signal (S13A Fig). We then examined the subcellular location of RAN1 using the transient expression system to express RAN1-GFP and WAK2-mCherry and we observed that RAN1-GFP was localized to the endomembrane and also was co-localized with the ER marker (S13B Fig). Further we wondered whether ATX1 and RAN1 are co-localized in a cell. To address this question, we used the transient expression system to express RAN1-GFP and ATX1-RFP, and we observed both proteins were co-localized on the ER membrane (Fig 5C). Together, our sub-cellular observations indicate both ATX1 and RAN1 can adhere to ER membranes.

To explore the possibility of ATX1 and RAN1 interacting *in planta*, we first carried out yeast two-hybrid experiments. ATX1 was fused to a GAL4 DNA-binding domain (BD) and RAN1-N (289 amino-terminal amino acids of RAN1) was ligated to a GAL4 activation domain (AD). Three days after these co-transformed yeast cells were grown on a synthetic dropout (-Leu/-Trp/-His/-Ade) medium with X-α-Gal (50 mg/L), we observed clones became blue, suggesting an interaction between ATX1 and RAN1-N in yeast cells (Fig 5D). This was next confirmed by using bimolecular fluorescence complementation (BiFC) assays in *Nicotiana benthamiana* leaves. For this, 35S: cLUC-ATX1 and 35S: nLUC-RAN-N constructs were made and co-transformed into the plants, and the results showed that these proteins interact with each other (Fig 5E). The co-immunoprecipitation (Co-IP) assays using ATX1-GFP/FLAG and RAN1-N-FLAG/GFP also showed that ATX1 interacts with RAN1 (Fig 5F). These results indicate that ATX1 physically interacts with RAN1 *in planta*. Together, the above data supports evidences for the hypothesis that the copper ions required for ethylene receptor biogenesis and signaling are transported through ATX1 to RAN1 and finally to ethylene receptors.

## Discussion

The functional characterization of genes using genetic approaches in plants depends on observable phenotypes when the gene being studied is mutated. For ethylene signaling, this mutational approach has long been saturated. It is important and pressing to overcome this problem. Recently developed chemical genetics approaches using specific active small molecules provide reversible, time and strength controllable genetic perturbations for genetic research and gene function characterization [36]. In this research, we applied a high-throughput plant chemical genetics screening and uncovered triplin, a novel small molecule, which cause a triple response in dark-grown *Arabidopsis* seedlings. Further we demonstrated triplin is a novel copper ion chelator and it alleviates the toxic effects of high copper ion levels on root growth. Additionally, the triple response phenotype resulting from triplin treatment is reduced by adding Cu^2+^ and Ag^+^. This is very different from another ion chelator neocuproine. Although neocuproine causes a triple response phenotype, this effect is suppressed by Zn^2+^ but not Cu^2+^. There is some white precipitate when we mixed Zn^2+^ and neocuproine (S7 Fig). While low concentration of copper ions could partially restore the hypersensitivity of *ran1-2* to neocuproine (S10B Fig). Another problem with neocuproine is that adding it to plant growth medium exaggerates the toxic effects of high levels of copper ions on plant root growth (S10C Fig). So we speculate that neocuproine could cause triple response phenotype by chelating copper ions like triplin, but its specificity and safety are questionable. Other copper ion chelators such as BCS do not elicit the triple response phenotype. We believe that triplin not only chelates copper ions in the plant growth medium, but also chelates copper ions in plant cells once it enters into plant cells. We predict that this restricts the access of copper ions needed for ethylene receptor biogenesis, and this condition lead to the triple response phenotype. Our study shows that triplin is different from other characterized copper chelators and it has the potential to perturb copper ion transport and to dissect the ethylene signaling network.

We still know little about the chemistry of triplin. One thing to note is triplin does not affect ethylene binding to ETR1 directly (Fig 4D). This may be related to its limited solubility in water and we noticed its precipitation was enhanced by adding CuSO_4_ or AgNO_3_ (S11C Fig). One possibility is its low solubility inhibits triplin to affect ethylene binding to ETR1 directly. However triplin could enter growing yeast cells expressing ETR1 when it was added to the yeast growing medium in which triplin decreased the copper level of yeast cells and affected ETR1 biosynthesis. This possibly resulted in producing abnormal ethylene receptors which could not bind ethylene properly (Fig 4D). It is important to characterize triplin in term of its chemistry in future.

Copper ion transport from the copper transporter RAN1 to the ethylene receptors for normal receptor biogenesis and function has been proposed, but many important details in this process are still missing [12–14]. For example, what component provides copper ion to RAN1 and where does this copper ion relay occur? These are some important questions we need to address in copper ion transport coupled ethylene receptor biogenesis and functioning. Our results examining the effects of triplin on known ethylene-related mutants indicates that triplin acts upstream of the receptors but does not affect ethylene biosynthesis. For example, *etr1-1* and *ein2* are both resistant to triplin. Furthermore, a triplin hypersensitive mutant screening identified both *ran1* and *atx1* mutants which are posited to function upstream of the receptors to deliver copper for normal receptor biogenesis. The hypersensitivity of both the *ran1* and *axt1* mutants was abolished by the mutations in downstream ethylene signaling network components such as *etr1-1* (Fig 2C and S11C Fig). We have noticed that *atx1-1* and *atx1-2* are less sensitive to triplin than *ran1-1* and *ran1-2* (Fig 5B), and the similar results were observed when treated with neocuproine (S10D Fig). These results suggest ATX1 may be positioned in the network prior to RAN1. Our ethylene binding assays indicate that triplin is not directly affecting the ethylene receptors, but appears to be affecting delivery of copper ions to the receptors. This supports the hypothesis that triplin targets copper ion transport upstream of the receptors.

Although RAN1 has been proposed to play a key role in copper ion transport in ethylene receptor biogenesis and signaling, the subcellular location of RAN1 has not been determined [12, 27]. The function of the well-known copper ion chaperone ATX1 is difficult to study by using conventional genetics approaches, since mutants such as *atx1-1* do not show a phenotype related to ethylene [25]. By taking advantage of triplin, ethylene signaling mutants and manipulating the concentration of copper ions in the plant growth medium, we provided genetic evidence for the first time of the interaction between RAN1 and ATX1, which contributes to transport copper ions to the ethylene receptors. ATX1 localizes widely in cells, indicating that it likely functions to transport copper ions to other targets in addition to RAN1. This is consistent with the report that ATX1 is essential for copper homeostasis. For example, it may act as a copper-buffer in plant cells [24, 25]. The other copper chaperone in *Arabidopsis* is CCH, but *cch* mutants have no ethylene related phenotypes. Additionally, we found that *cch* mutants had wild type responses to triplin suggesting that CCH is not important for delivery of copper ions to RAN1 and eventually, the ethylene receptors. The subcellular localization of RAN1 was not identified previously, but based on the phenotypes of its mutants it seems likely that RAN1 is active in an endomembrane system compartment, perhaps the ER [12, 27]. Our microscopic observations using ATX1, RAN1, and organelle markers, as well as the results of molecule interaction assays *in planta* indicate that RAN1 and ATX1 are partially co-localized and interact on the ER. This supports the idea that ATX1 and RAN1 interact as part of copper transport to the ethylene receptors.

Copper ions are important to human health where its disorder can result in various diseases. More and more reports show that copper disorders are closely related to human Alzheimer's disease and cancers. Therefore various copper chelators have been identified and characterized in order to cure these important human diseases [37–40]. Although a few copper ion chelators have been used in therapy, thus far their mechanisms of action are not clear [41]. One problem using these chelators is they are not very specific for copper ions [42], and some chelators, such as neocuproine, might enhance the toxic effects of copper ions on the organism being treated as we show in this study for plant growth. To this end, triplin is a unique copper ion chelator that may provide a new lead structure for copper ion chelators related to developing drug. Thus, the model plant *Arabidopsis* could be another useful platform to carry out studies to uncover copper ion chelator and explore its action mechanism *in vivo*.

## Materials and methods

### Plant chemical genetics screenings

The plant chemical genetics screenings using dark-grown *Arabidopsis* seedlings against a structure novel and diverse synthetic chemical library of 12,000 small molecules from Life Chemicals Inc was performed as previously reported [32, 33]. For all screenings, surface-sterilized *Arabidopsis* Col-0 seeds suspended in 0.1% agar were evenly distributed into 96-well plates that contained 0.8% agar, 0.3xMS salts(Sigma-Aldrich), 100 μM individual chemical per well and 1% DMSO (carrier solvent). Seeds were stratified for 3 days in a 4 C refrigerator, transferred to day-light for 1–4 hours then transferred in dark to grow for 3 days at 22 C in a light-tight growth cabinet. Seedlings phenotypes were recorded and imaged using a SZX16 dissecting microscope. Chemicals that caused dark-grown Col-0 seedlings triple response like phenotypes were retested. For each active chemical, its dose effect on the length of the hypocotyls or roots of assayed seedlings were measured using Image J (NIH).

### Isolation and characterization of triplin resistant mutants

To acquire triplin resistant mutant, we used a mutant screening strategy that is modified from [30]. Briefly 20,000 M2 seeds from 5,000 ethylmethane sulfonate (EMS)-mutagenized M1 Col-0 plants were surface-sterilized and grown under the same growth conditions and chemical dose as the plant chemical genetics screenings. The phenotypes of seedlings were examined under the microscope, and these with longer hypocotyl or without exaggerative apical hooks were considered as putative triplin resistant mutants, and all these mutants were then retested for their chemical genetics phenotypes in the next generation.

### Plant ethylene measurement

Ethylene measurements on plants were performed as previously described [43, 44]. Col-0 seeds were surface sterilized and planted on 0.5xMS solid medium. After stratification at 4 C for 3 days, plates were exposed to light for 1–4 hours and then transferred in dark to grow for 2 days at 22 C. After seed germination, every 22 seedlings per group (n = 3) were transferred to a gas chromatography vial with half volume 0.5xMS growth medium contain 100 μM triplin, 50 μM ACC or 1%DMSO and incubated for 3 days under continual dark. Accumulated ethylene was measured by gas chromatography (Agilent Technologies, 6890N Network GC System) [44]. Seedlings weight is fresh weight.

### Plant copper ion content assay

Three-day-old etiolated Col-0 seedlings grown on 0.5xMS solid medium with 20 μM CuSO_4_ and/or 100 μM triplin were collected. The copper ion contents in plants were then determined by inductively coupled plasma-mass spectrometry as previously described [45].

### MALDI-TOF-MS analysis

For these assays, 100 μl of the metal ions (100 μM or 10 mM) and 100 μl triplin (100 μM or10 mM) were mixed for MALDI-TOF-MS analysis according to [46]. A time-of–flight Axima Performance mass spectrometer (Shimadzu, Japan) was used. All mass spectra in this work were acquired at positive ion reflection mode. The data were controlled by a software application of MALDI-MS. The matrix is 5 mg/ml α-Cyano-4-hydroxycinnamic acid (CHCA). Mass spectrum scanning range is 400-1000Da.

### Ethylene binding assays

For this, we expressed the first 128 amino acids of ETR1 containing the ethylene binding domain as a fusion protein to GST (glutathione *S-*transferase) (ETR1 [1–128]-GST) in *Pichia pastoris* as previously described [47]. Binding assays were then conducted on either intact yeast cells or isolated membranes as previously described using a radioligand binding assay [48, 49]. Briefly, to test the effects of triplin on ethylene binding to its receptors in intact yeast cells, yeast were incubated at 30°C in the presence or absence of 100 μM triplin for 3 days. Intact yeast cells were then harvested and assayed for binding. For binding assays to membranes, cells were disrupted and membranes isolated as previously described [11] and ethylene binding determined in the presence or absence of 100 μM triplin. Saturable ethylene binding is indicated as counts per minute (CPM) and was calculated by subtracting the amount of radioactivity bound in the presence of excess non-radioactive ethylene from what was bound in the absence of unlabeled ethylene. We used western blots with anti-GST antibodies to ensure equal levels of ETR1 [1–128]-GST [11].

### Constructs and plant transformation

The constructs and plant transformation were performed as previously described [32]. The coding sequence (CDS) of ATX1 (AT1G66240) and RAN1 (AT5G44790) was amplified from cDNA of Col-0 by PCR using primers listed in S2 Table. Fragments were then cloned into the entry vector pDONR-zeocin by BP reactions by following the instructions of the manufacturer (Life Technology, USA). Then genes were introduced to different pGWB vectors by LR reactions. The vectors were then used to transform *Agrobacterium* strain GV3101, the transformed agrobacteria were finally used to transform flowering *Arabidopsis* plants via the floral-dip method [50].

### Quantitative real-time RT-PCR (qRT-PCR)

The qRT-PCR was done as previously described [32]. For testing the expression level of *ERF1*, seeds were surface sterilized and planted on 0.5xMS growth medium contain different chemicals in petri dish plates, after stratification at 4 C for 3 days, plates were exposed to light for 1–4 hours then transferred to dark for 3 days (22 C), and seedlings were collected. For testing the expression level of *RAN1* in wild type and transgenic plants, 3-week old plant leaves were collected and total RNAs were extracted and used to synthesize the cDNAs by reverse transcription. Primers for *ERF1* were made according to [43]. All primers used were listed in S2 Table. The *ACTIN* gene was amplified and used as an internal control.

### Confocal observation and imaging

pDONR-ATX1 and pDONR-RAN1 were introduced into pGWB vectors as described in [32]. The construct combinations of ATX1:PGWB5/PGWB654 and RAN1:PGWB605 were used to transform *Agrobacterium* GV3101; then the *Agrobacterium* were infiltrated into leaves of N. benthamiana with P19 [32]. After 2-day incubation, the transformed plant leaves were observed and imaged under a confocal microscope (Olympus FV1000). For transgenic plants, the 5-day-old light grown roots or 3-day-old dark-grown hypocotyls were imaged under the confocal microscope. However photos in S12C and S12D Fig were imaged by NIKON A1R. ER, Golgi and plasma membrane associated protein markers used is AtWAK2, GmMAN1 and OsREM4.1 respectively [51, 52]. *Arabidopsis* WAK2-mCherry or GmMAN1-mCherry transgenic seeds are gifts from Chi-Kuang Wen lab of Shanghai Institute of Plant Physiology and Ecology, Chinese Academy of Sciences. For DAPI staining, seedlings were fixed in 5% methanol, immersed in 2 μg/ml DAPI in phosphate buffer saline (PBS) and viewed by fluorescence microscopy under UV light.

### Yeast two-hybrid assay

Yeast two-hybrid experiments were performed as previously described [32]. pGADT7 (RAN1 N terminal) and pGBKT7 (ATX1) were transformed simultaneously into the yeast strain AH109, the colonies were transferred to a SD (-Leu/-Trp/-His-Ade) solid medium with X-α-gal according to the manufacturer’s protocols (Clontech) for 3 days. The positive clones were identified because they grew well and became blue.

### BiFC and Co-IP in *N. benthamiana*

BiFC and Co-IP assays were performed as previously described [32, 52]. The Split-Luciferase Complementation system was used. The construct combinations of cLuc-ATX1/nLuc-RAN1-N were used to transform *Agrobacterium* GV3101; then the *Agrobacterium* were infiltrated into leaves of *N*. *benthamiana* with P19 as previously described. After 2-day incubation, 1 mM luciferin (Sigma) was filtrated into the leaves and the pictures were recorded using a CCD imaging system (Berthold, https://www.berthold.com/). For the Co-IP assays, the N. benthamiana leaves were transfected with *Agrobacterium* containing ATX1-GFP/FLAG and RAN1-N-FLAG/GFP, and incubated for 2 days. Total proteins were extracted with extraction buffer [50 mM Tris-HCl, pH 7.5, 150 mM NaCl, 5 mM MgCl_2_, 1 mM EDTA, 1% Triton X-100, and 0.1% protease inhibitor cocktail (Promega)] for 20 minutes, and centrifuged at 4°C at 12,000 rpm for 20 min. The supernatant was incubated with pretreated anti-FLAG antibody coupled agarose beads (Abmart) for 2 hours at 4°C. Beads were washed three times with wash buffer (50 mM Tris-HCl, pH 7.5, 150 mM NaCl, 5 mM MgCl_2_, 1 mM EDTA, 1% Triton X-100). The bound proteins were eluted with 2xSDS loading buffer and boiled at 100°C for 5 minutes. The eluted proteins were separated on SDS-PAGE and immunoblotted with anti-FLAG antibody (Abmart) and anti-GFP antibody (Abmart).

### Accession numbers

ATX1 (AT1G66240), RAN1 (AT5G44790), CCH (AT3G56240), ETR1 (AT1G66340), EIN2 (AT5G03280), EIN3 (AT3G20770), Triplin (F0655-1171)

## Supporting information

S1 Fig Other chemicals that cause a triple response phenotype uncovered in this study. The phenotypes of 3-day-old, dark-grown Col-0 and *ein2-5* seedlings treated with 100 μM F0617-0170, F0665-0601, F1806-0031 or F2001-0992. For AgNO_3_ treatment a concentration of 500 μM was used. Scale bars represent 1 mm.
(TIF)

S2 Fig Phenotypes of triplin resistant EMS mutants acquired in this research. (A) The phenotype of 3-day -old, dark-grown seedlings from the M2 generation of the triplin resistant dominant mutants *45–1* and *45–2* treated with 100 μM triplin. (B) The genome DNA sequencing raw data of mutants *45–1* and *45–2* showing the G194A substitution mutations identical as the mutation in *etr1-1*. (C) The phenotypes of 3-day-old, dark-grown recessive triplin resistant mutants *39–1*, *39–2*, *41–1*, *41–2*, *42*, *43* and *44* and their *F1*s with *ein2* treated with 100 μM triplin. For comparison, the phenotypes of Col-0 and *ein2* are shown. Scale bars represent 1 mm.
(TIF)

S3 Fig Ethylene insensitive mutants are resistant to triplin. The phenotypes of 3-day-old, dark-grown seedlings without (-) or with (+) 100 μM Triplin. The phenotypes of (A) Col-0, *ein2-5* and the *ein2-5* complementary lines, *com17-2* and *com27-7*, and (B) ethylene resistant mutants of ethylene receptors, *ers1-1*, *ers2-1*, *etr2-1* and *ein4*.Scale bars represent 1 mm.
(TIF)

S4 Fig Triplin does not enhance ethylene biosynthesis. (A) qRT-PCR analysis of the relative expression levels of *ACS5*, *ACS6* and *ACS11*. 3-day-old dark-grown Col-0 seedlings were treated with DMSO or 100 μM triplin. Each experiment was repeated three times, and error bars represent SEM. (B) Hypocotyl length of 3-day-old, dark-grown seedlings of Col-0 and *cin5-1* treated with DMSO or 100 μM triplin. Each experiments was repeated three times, more than 30 seedlings were used every time. Error bars represent SEM. No significant difference was observed by two-tailed Student’s t-test using 0.05 cut-off. (C) Hypocotyl length of 3-day-old, dark-grown Col-0 seedlings in the presence of triplin without or with 10 μM AVG. The experiments were repeated three times with similar results (n ≥ 30).Values represent means ± SD, and no significant difference was observed by two-tailed Student’s t-test using 0.05 cut-off. (D) Gas chromatography analysis of ethylene production in Col-0 treated by 100 μM triplin, 50 μM ACC, or 1% (v/v) DMSO as a control. n = 3; error bars represent SEM. The scale bars represent 1 mm.
(TIF)

S5 Fig The effect of different metal ions on triplin responses. (A) The phenotypes of 3-day-old, dark-grown Col-0 seedlings treated with 100 μM triplin in the presence of the indicated metal salts. The concentrations of metal salts were 200 μM except for NiSO4 where 100 μM was used. The scale bars represent 1 mm. (B) The hypocotyl length of the seedlings in (A). The experiments were repeated three times with similar results (n ≥ 30).Values represent means±SD., and no significant difference was observed using two-tailed Student’s t-test with 0.05 cut-off.
(TIF)

S6 Fig Triplin alleviates the copper ion toxic effect, and Ag^+^ could reverse the effects of triplin. (A) The phenotypes of 3-day-old, dark-grown Col-0 seedlings treated with 100 or 200 μM CuSO_4_ with or without 100 or 200 μM triplin. (B) The hypocotyl length of the seedlings in (A). Each experiments was repeated three times, more than 30 seedlings were used every time. Error bars represent SEM. *P < 0.05 (two-tailed Student’s t-test) indicated a significant difference between groups of different treatments. (C) The phenotypes of 3-day-old, dark-grown Col-0 seedlings treated with 100 μM triplin in the presence of 0, 100, 200, or 500 μM AgNO_3_. (D) The hypocotyl length of the seedlings in (C). All experiments were repeated three times with the similar results. Error bars represent SD (n > 30). In (A) and (C), the scale bars represent 1 mm.
(TIF)

S7 Fig The reaction of different metal ions and copper chelators. The appearance 100 μl of 100 mM metal salts of CaCl_2_, ZnSO_4_, CuSO_4_ or AgNO_3_ were mixed with 100 μl of 10mM copper ion chelators triplin, neocuproine or BCS in 1.5 ml Eppendof tubes. The scale bar represents 1 mm.
(TIF)

S8 Fig MALDI-TOF-MS analysis of the reaction products of metal ions and triplin. The MALDI-TOF-MS analysis results of the reaction product of 100 μM ZnSO_4_ and 100 μM triplin (A), the reaction product of 100 μM FeSO_4_ and 100 μM triplin (B) and the reaction product of 10 mM AgNO_3_ and 10 mM triplin (C). The metal salts and triplin were mixed as described in S7 Fig.
(TIF)

S9 Fig The hypersensitivity of seedling to triplin treatment grown in the growth mediun without copper ion is dependent on the ethylene signaling pathway. The phenotypes of 3-day-old, dark-grown *ein2-5* seedlings grown on different growth medium with or without 50 μM triplin. -Cu indicates the growth medium was made of plant essential elements except copper ion. BCS indicates the growth medium of 0.5xMS with 500 μM BCS. The scale bar represents 1 mm.
(TIF)

S10 Fig The effects of neocuproine on seedlings treated with different ions. (A) The phenotypes of 3-day-old, dark-grown Col-0 seedlings treated with 100 μM neocuproine in the presence of no metal, 500 μM AgNO_3_, 100 μM CuSO_4_ or 100 μM ZnSO_4_. For comparison, the phenotypes of *ein2-5* grown with only 100 μM neocuproine are shown. (B) The phenotypes of 3-day-old, dark-grown *ran1-2* seedlings grown on 5 μM neocuproine without or with 10 or 20 μM CuSO_4_ are shown. (C) The phenotypes of 3-day-old, dark-grown Col-0 seedlings without or with 5, 20, 50 or 100μM neocuproine coupled with 0, 10 or 20 μM CuSO_4_ are shown. (D) *atx1-1* and *ran1-2* are hypersensitive to neocuproine. The phenotypes of 3-day-old, dark-grown seedlings of Col-0, *atx1-1* and *ran1-2* were treated with 5, 10 or 20 μM neocuproine. In all pictures, the scale bars represent 1 mm.
(TIF)

S11 Fig The hypersensitivity of *atx1* to triplin depends on the ethylene signaling pathway. (A) A diagram shows the locations of the *ATX1* T-DNA insertion mutants, Salk_026221 (*atx1-1*) and Salk_041022 (*atx1-2*). (B) The gene expression of the *atx1-1* and *atx1-2* were examined by RT-PCR. *ACTIN* was used as a loading control. (C) The phenotypes of 3-day-old, dark-grown seedlings of *atx1-1*, *atx1-1 etr1-1* and *atx1-1 ein2-5* treated with 100 μM triplin. The scale bar represents 1 mm. (D) The phenotypes of 3-day-old, dark-grown seedlings of Col-0 and *atx1-1* treated with 20 μM triplin with (+) or without (-) 10μM CuSO_4_. The scale bars represent 1 mm.
(TIF)

S12 Fig ATX1 localizes at cytoplasm and nucleus. (A) Scanning images of the roots of *35S*:*ATX1-GFP (atx1-1)* seedlings under a confocal microscope (Olympus FV1000). (B) Scanning images of the roots of *35S*:*ATX1-GFP (atx1-1)* seedlings after DAPI staining under a confocal microscope (Olympus FV1000). (C) Scanning images of the epidermal cells of the hypocotyl of *35S*:*ATX1-GFP (atx1-1)* seedling by a confocal microscope (NIKON A1R). (D) Scanning image of the bottom of a cell in the hypocotyl of *35S*:*ATX1-GFP (atx1-1)* seedling by a confocal microscope (NIKON A1R). (E) Scanning images of the hypocotyls of *F1* from *35S*:*ATX1-GFP (atx1-1)* and transgenic marker lines with WAK2-mCherry under a confocal microscope (Olympus FV1000). (F) Scanning images of the hypocotyls of *F1* from *35S*:*ATX1-GFP (atx1-1)* and transgenic marker lines with GmMAN1-mCherry under a confocal microscope (Olympus FV1000). All scale bars represent 20 μm.
(TIF)

S13 Fig ATX1 and RAN1 co-localized with ER membrane marker in N. benthamiana leaves. (A) ATX1-GFP and WAK2 / GmMAN1 / OsREM4.1-mCherry were transiently expressed in N. benthamiana leaves and their co-localizations were shown. (B) RAN1-GFP and WAK2 / GmMAN1 / OsREM4.1-mCherry were transiently expressed in N. benthamiana leaves and their co-localizations were shown. All images were made under a confocal microscope (Olympus FV1000).Scale bars represent 20 μM.
(TIF)

S14 Fig Overexpression of *RAN1* results in plant resistance to triplin. (A) The phenotypes of 3-day-old, dark-grown seedlings of Col-0 and two *35S*:*RAN1-GFP* transgenic lines treated with 0, 30, 50 or 100 μM triplin. (B) qRT-PCR analysis of the relative *RAN1* expression levels in two *35S*:*RAN1-GFP* lines. Each experiment was repeated three times, and error bars represent SEM.
(TIF)

S1 Table Structure of triplin like chemicals. (DOCX)

S2 Table Primers used in this research. (DOCX)
