# Supplementary figures and images for: Triplin, a small molecule, reveals copper ion transport in ethylene signaling from ATX1 to RAN1
Source: PLoS Genet. 2017 Apr 7;13(4):e1006703. doi: 10.1371/journal.pgen.1006703 (PMC5400275; doi:10.1371/journal.pgen.1006703)

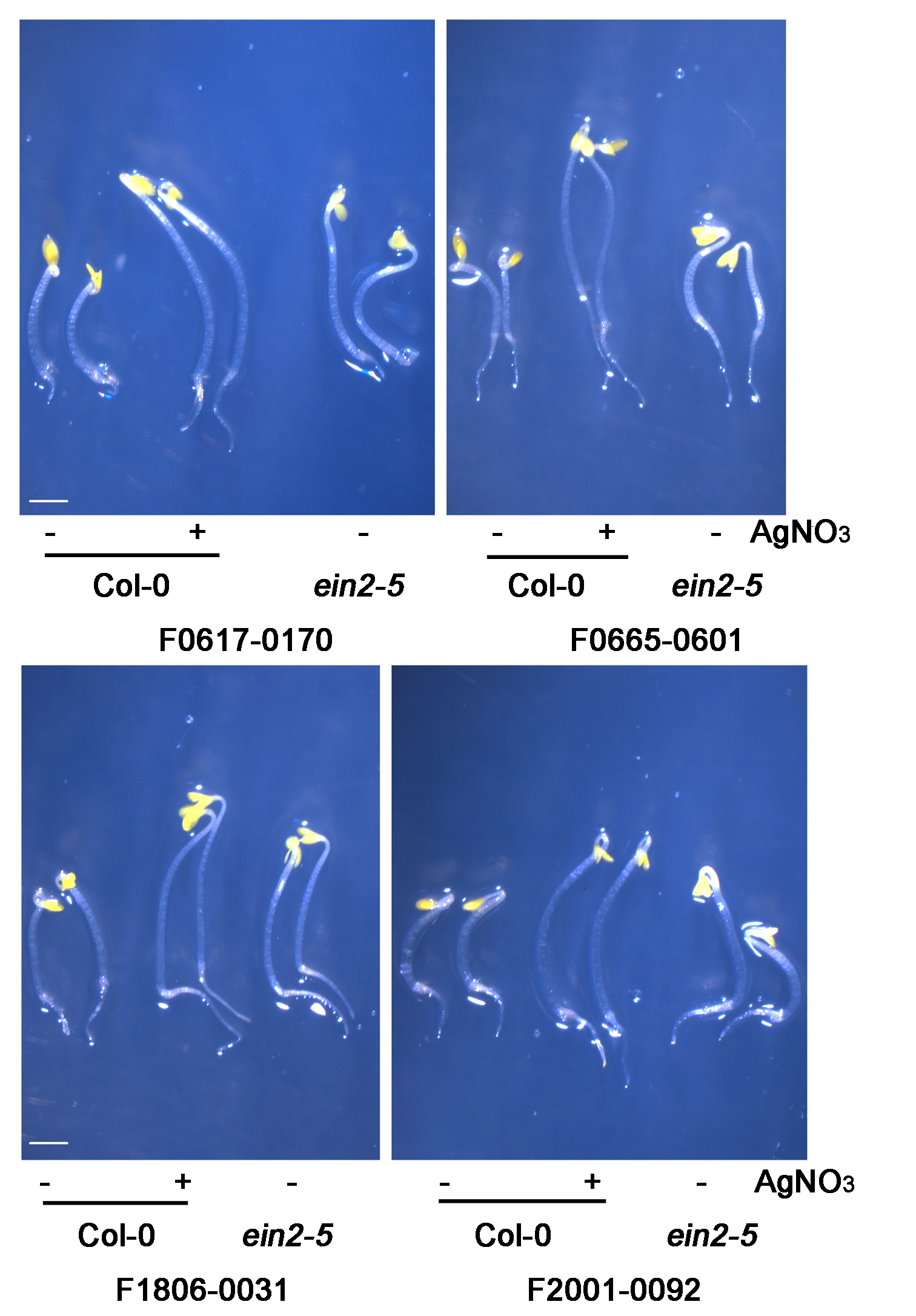

Supplement: S1 Fig — The phenotypes of 3-day-old, dark-grown Col-0 and ein2-5 seedlings treated with 100 μM F0617-0170, F0665-0601, F1806-0031 or F2001-0992. For AgNO3 treatment a concentration of 500 μM was used. Scale bars represent 1 mm. (TIF) [file pgen.1006703.s001.tif]

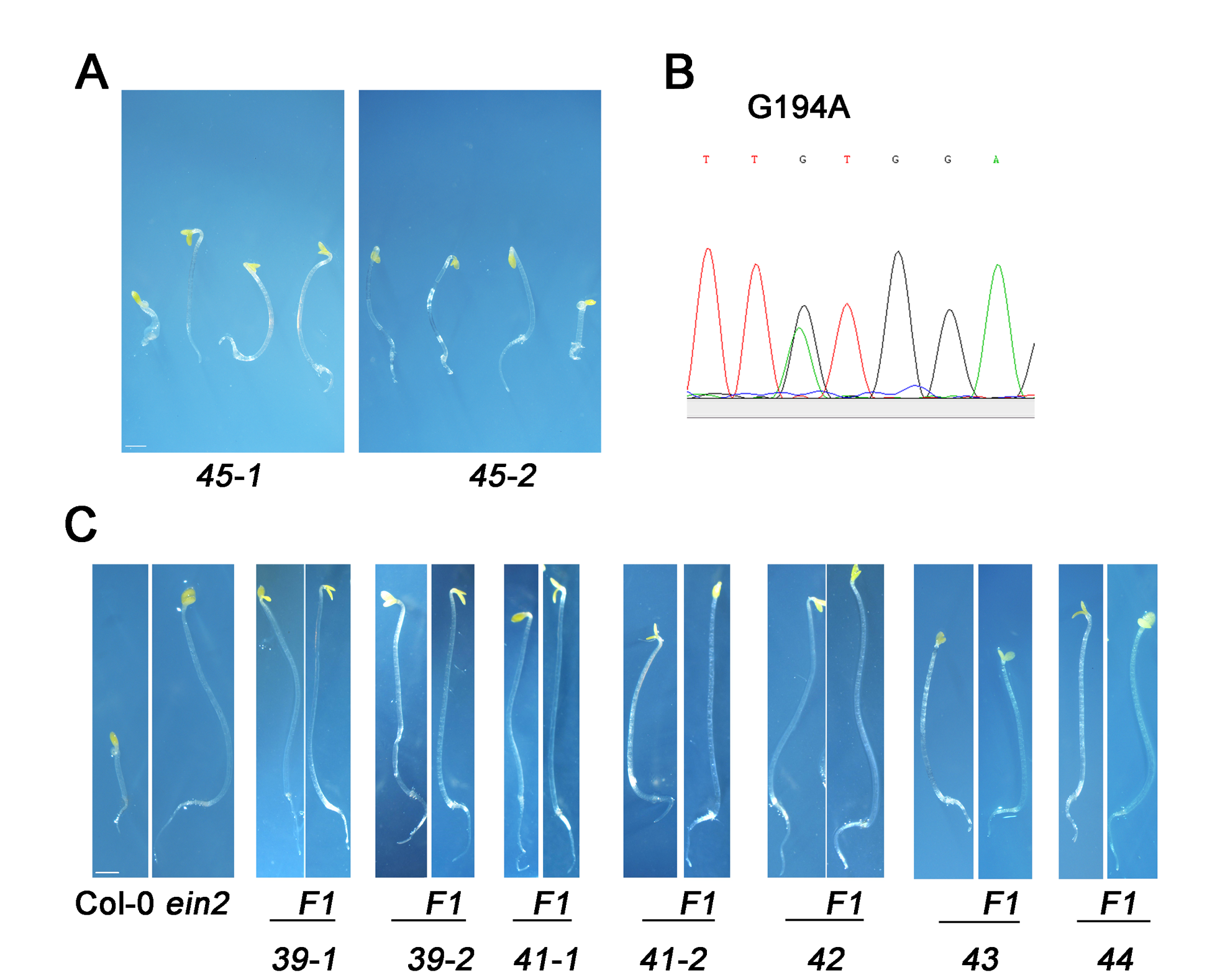

Supplement: S2 Fig — (A) The phenotype of 3-day -old, dark-grown seedlings from the M2 generation of the triplin resistant dominant mutants 45–1 and 45–2 treated with 100 μM triplin. (B) The genome DNA sequencing raw data of mutants 45–1 and 45–2 showing the G194A substitution mutations identical as the mutation in etr1-1. (C) The phenotypes of 3-day-old, dark-grown recessive triplin resistant mutants 39–1, 39–2, 41–1, 41–2, 42, 43 and 44 and their F1s with ein2 treated with 100 μM triplin. For comparison, the phenotypes of Col-0 and ein2 are shown. Scale bars represent 1 mm. (TIF) [file pgen.1006703.s002.tif]

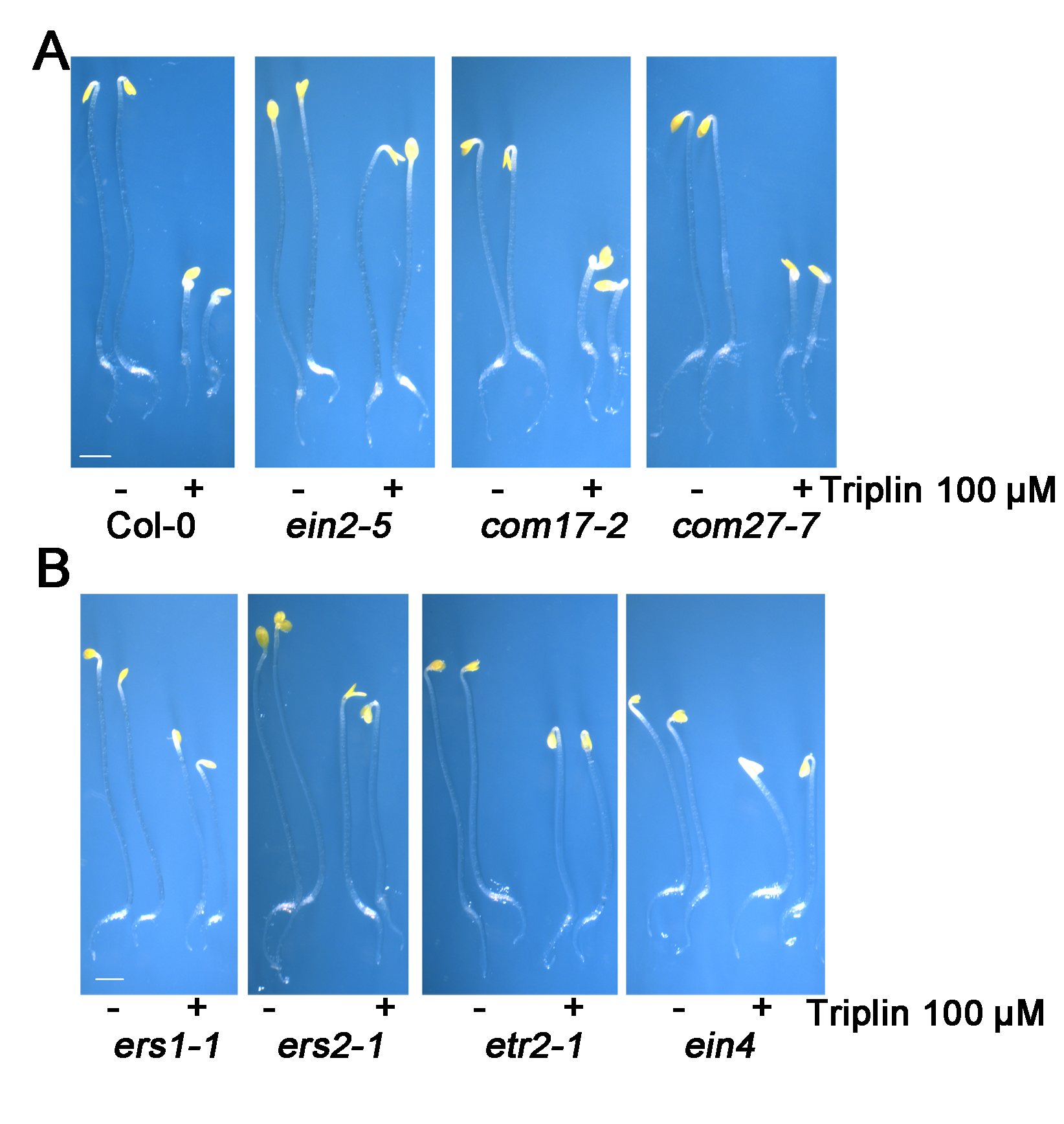

Supplement: S3 Fig — The phenotypes of 3-day-old, dark-grown seedlings without (-) or with (+) 100 μM Triplin. The phenotypes of (A) Col-0, ein2-5 and the ein2-5 complementary lines, com17-2 and com27-7, and (B) ethylene resistant mutants of ethylene receptors, ers1-1, ers2-1, etr2-1 and ein4.Scale bars represent 1 mm. (TIF) [file pgen.1006703.s003.tif]

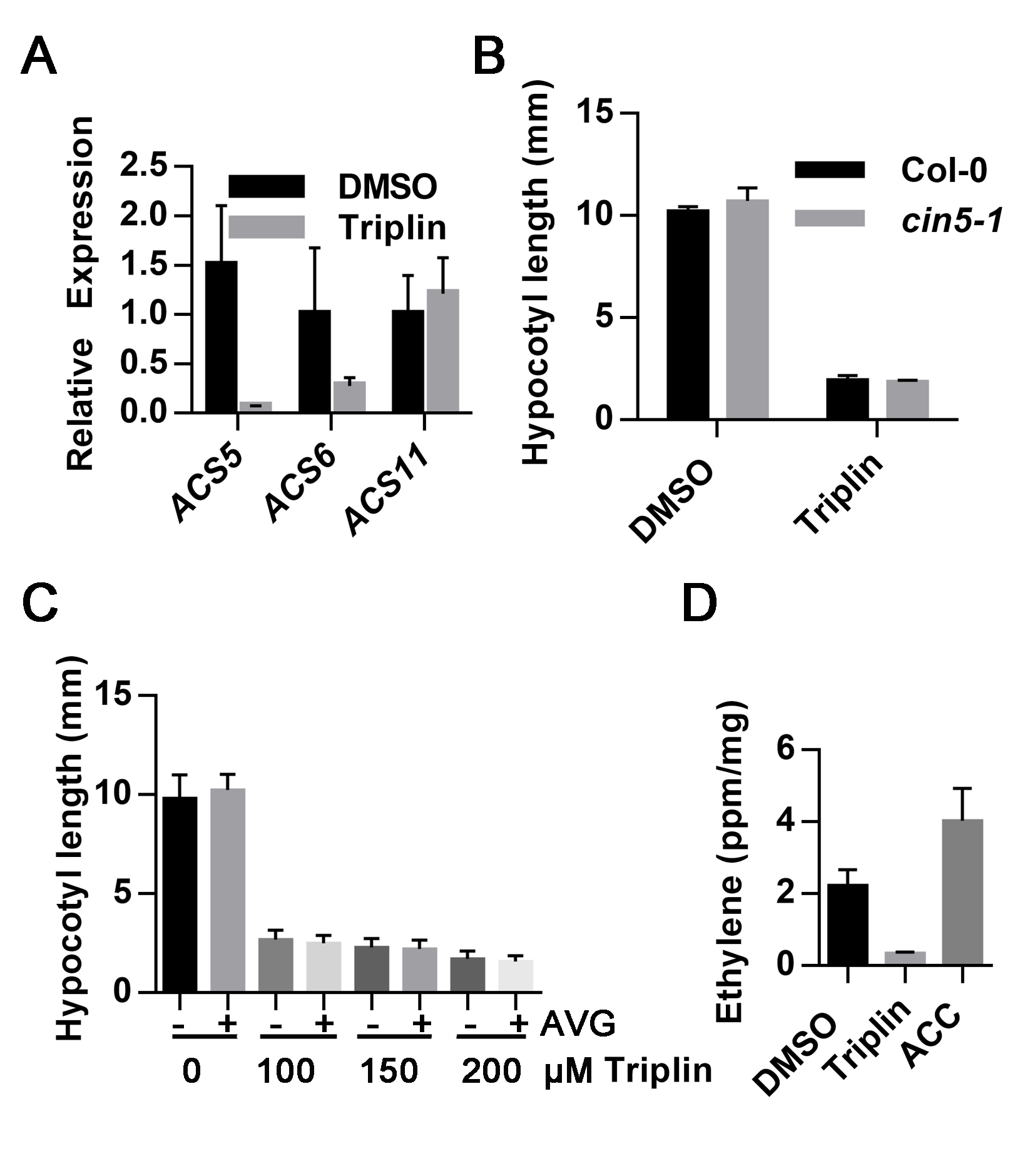

Supplement: S4 Fig — (A) qRT-PCR analysis of the relative expression levels of ACS5, ACS6 and ACS11. 3-day-old dark-grown Col-0 seedlings were treated with DMSO or 100 μM triplin. Each experiment was repeated three times, and error bars represent SEM. (B) Hypocotyl length of 3-day-old, dark-grown seedlings of Col-0 and cin5-1 treated with DMSO or 100 μM triplin. Each experiments was repeated three times, more than 30 seedlings were used every time. Error bars represent SEM. No significant difference was observed by two-tailed Student’s t-test using 0.05 cut-off. (C) Hypocotyl length of 3-day-old, dark-grown Col-0 seedlings in the presence of triplin without or with 10 μM AVG. The experiments were repeated three times with similar results (n ≥ 30).Values represent means ± SD, and no significant difference was observed by two-tailed Student’s t-test using 0.05 cut-off. (D) Gas chromatography analysis of ethylene production in Col-0 treated by 100 μM triplin, 50 μM ACC, or 1% (v/v) DMSO as a control. n = 3; error bars represent SEM. The scale bars represent 1 mm. (TIF) [file pgen.1006703.s004.tif]

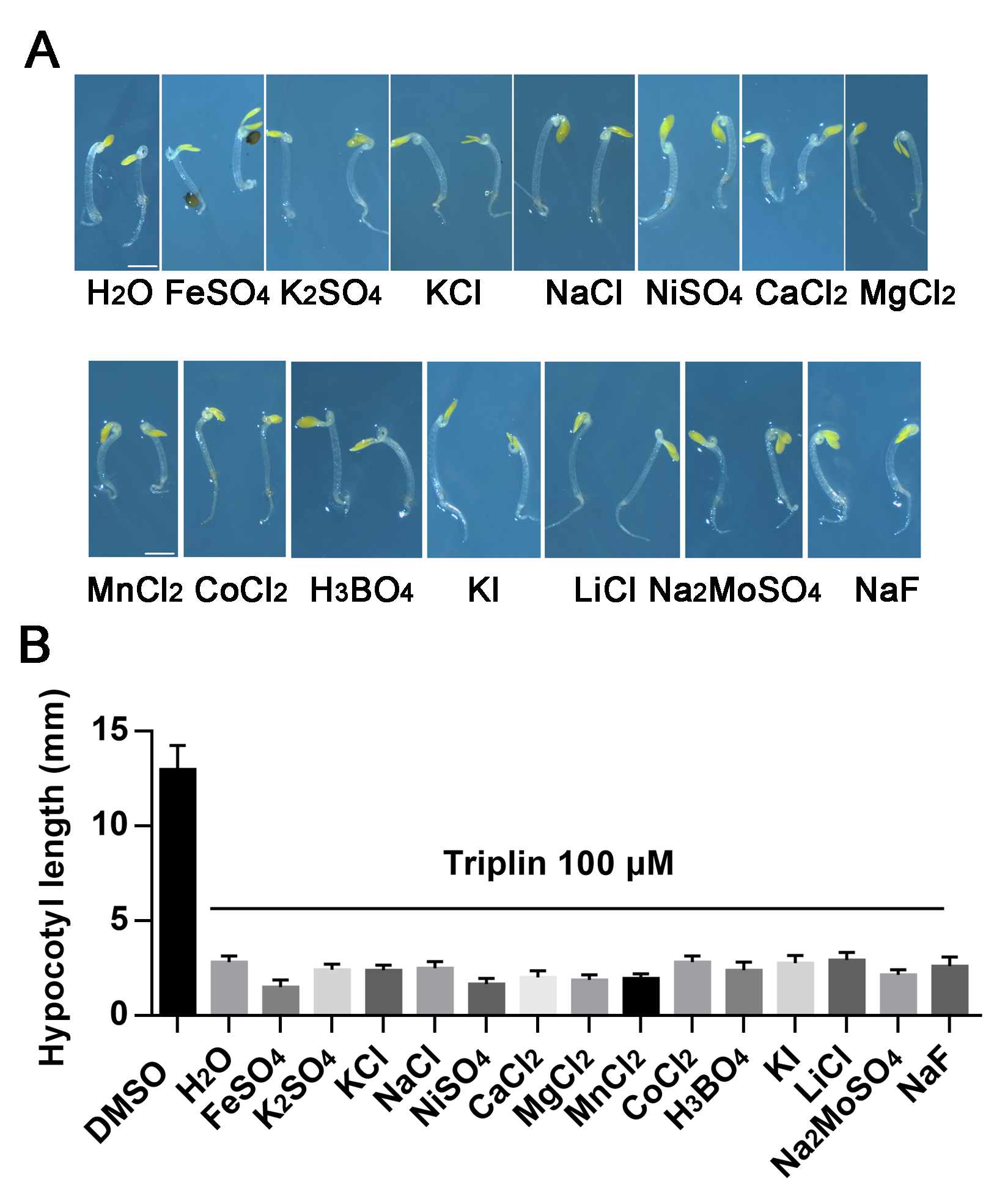

Supplement: S5 Fig — (A) The phenotypes of 3-day-old, dark-grown Col-0 seedlings treated with 100 μM triplin in the presence of the indicated metal salts. The concentrations of metal salts were 200 μM except for NiSO4 where 100 μM was used. The scale bars represent 1 mm. (B) The hypocotyl length of the seedlings in (A). The experiments were repeated three times with similar results (n ≥ 30).Values represent means±SD., and no significant difference was observed using two-tailed Student’s t-test with 0.05 cut-off. (TIF) [file pgen.1006703.s005.tif]

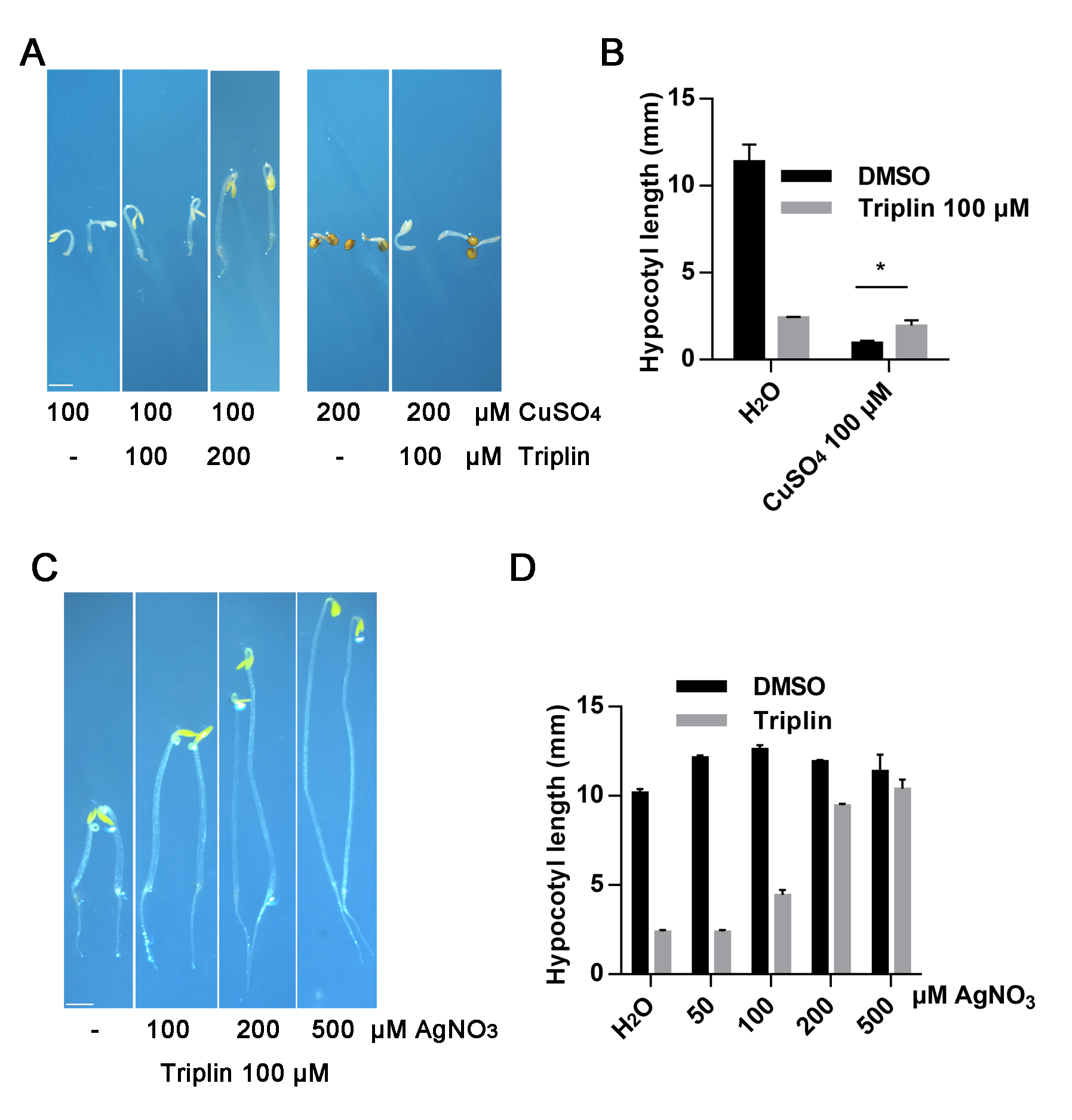

Supplement: S6 Fig — (A) The phenotypes of 3-day-old, dark-grown Col-0 seedlings treated with 100 or 200 μM CuSO4 with or without 100 or 200 μM triplin. (B) The hypocotyl length of the seedlings in (A). Each experiments was repeated three times, more than 30 seedlings were used every time. Error bars represent SEM. *P < 0.05 (two-tailed Student’s t-test) indicated a significant difference between groups of different treatments. (C) The phenotypes of 3-day-old, dark-grown Col-0 seedlings treated with 100 μM triplin in the presence of 0, 100, 200, or 500 μM AgNO3. (D) The hypocotyl length of the seedlings in (C). All experiments were repeated three times with the similar results. Error bars represent SD (n > 30). In (A) and (C), the scale bars represent 1 mm. (TIF) [file pgen.1006703.s006.tif]

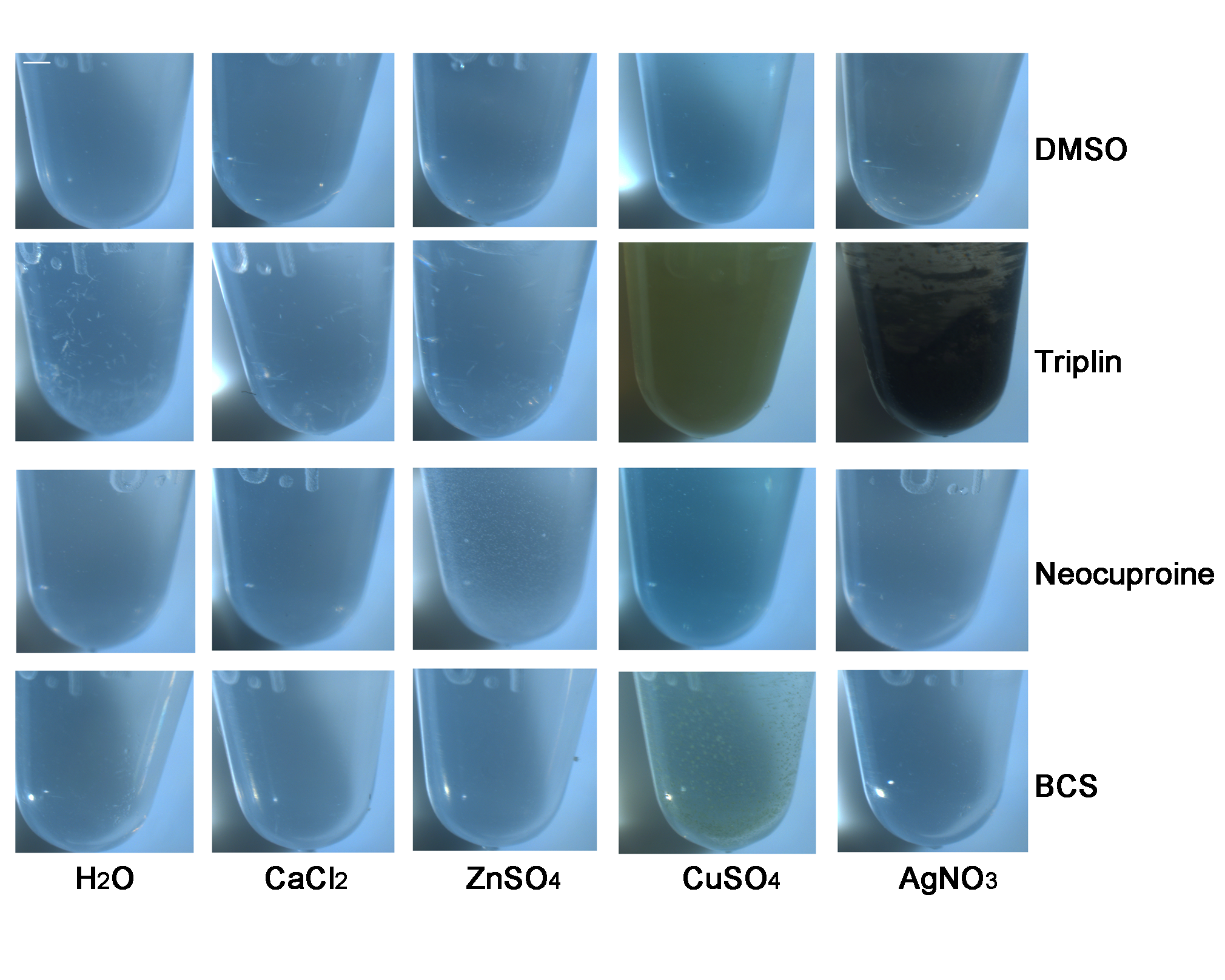

Supplement: S7 Fig — The appearance 100 μl of 100 mM metal salts of CaCl2, ZnSO4, CuSO4 or AgNO3 were mixed with 100 μl of 10mM copper ion chelators triplin, neocuproine or BCS in 1.5 ml Eppendof tubes. The scale bar represents 1 mm. (TIF) [file pgen.1006703.s007.tif]

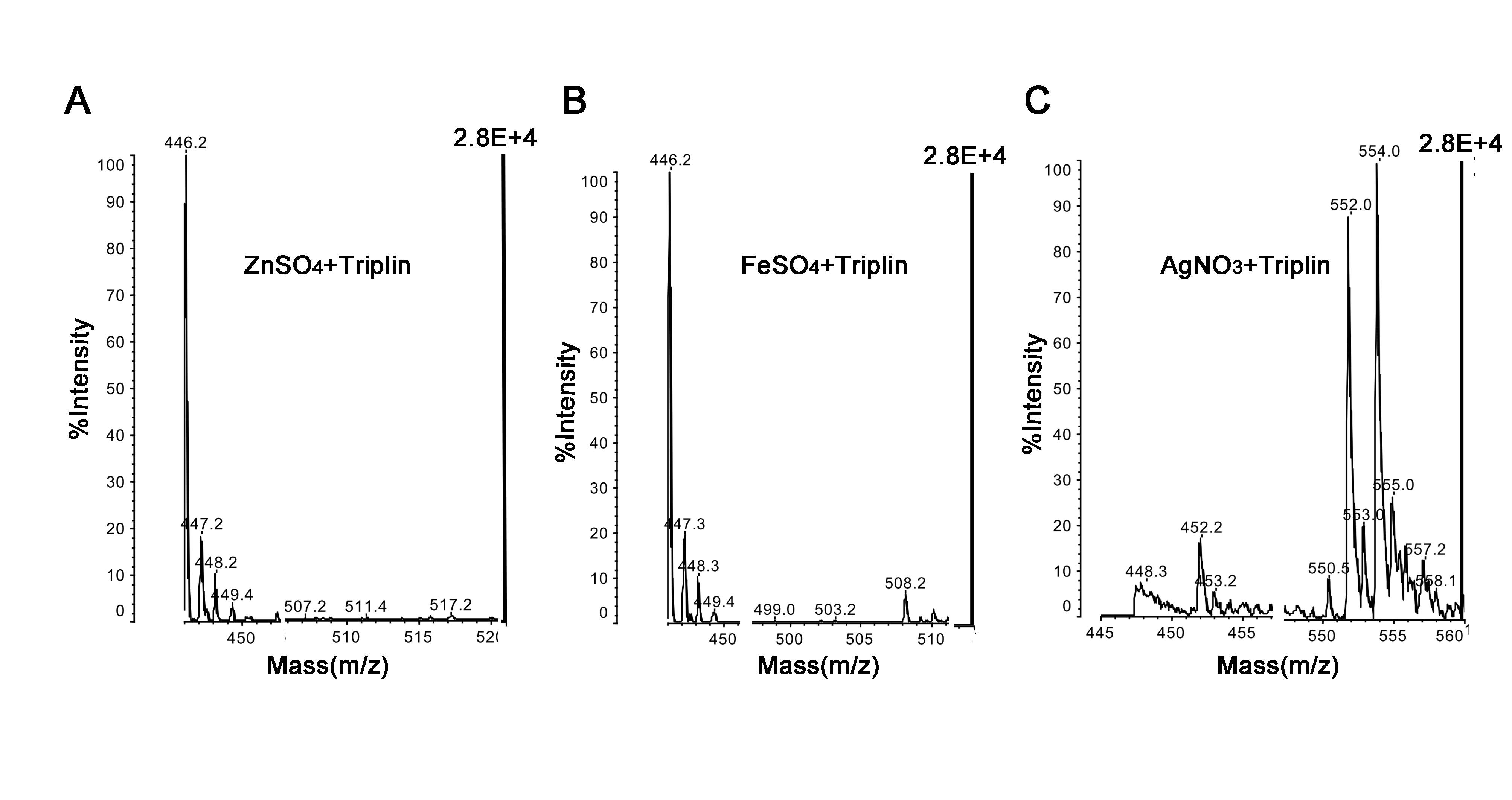

Supplement: S8 Fig — The MALDI-TOF-MS analysis results of the reaction product of 100 μM ZnSO4 and 100 μM triplin (A), the reaction product of 100 μM FeSO4 and 100 μM triplin (B) and the reaction product of 10 mM AgNO3 and 10 mM triplin (C). The metal salts and triplin were mixed as described in S7 Fig. (TIF) [file pgen.1006703.s008.tif]

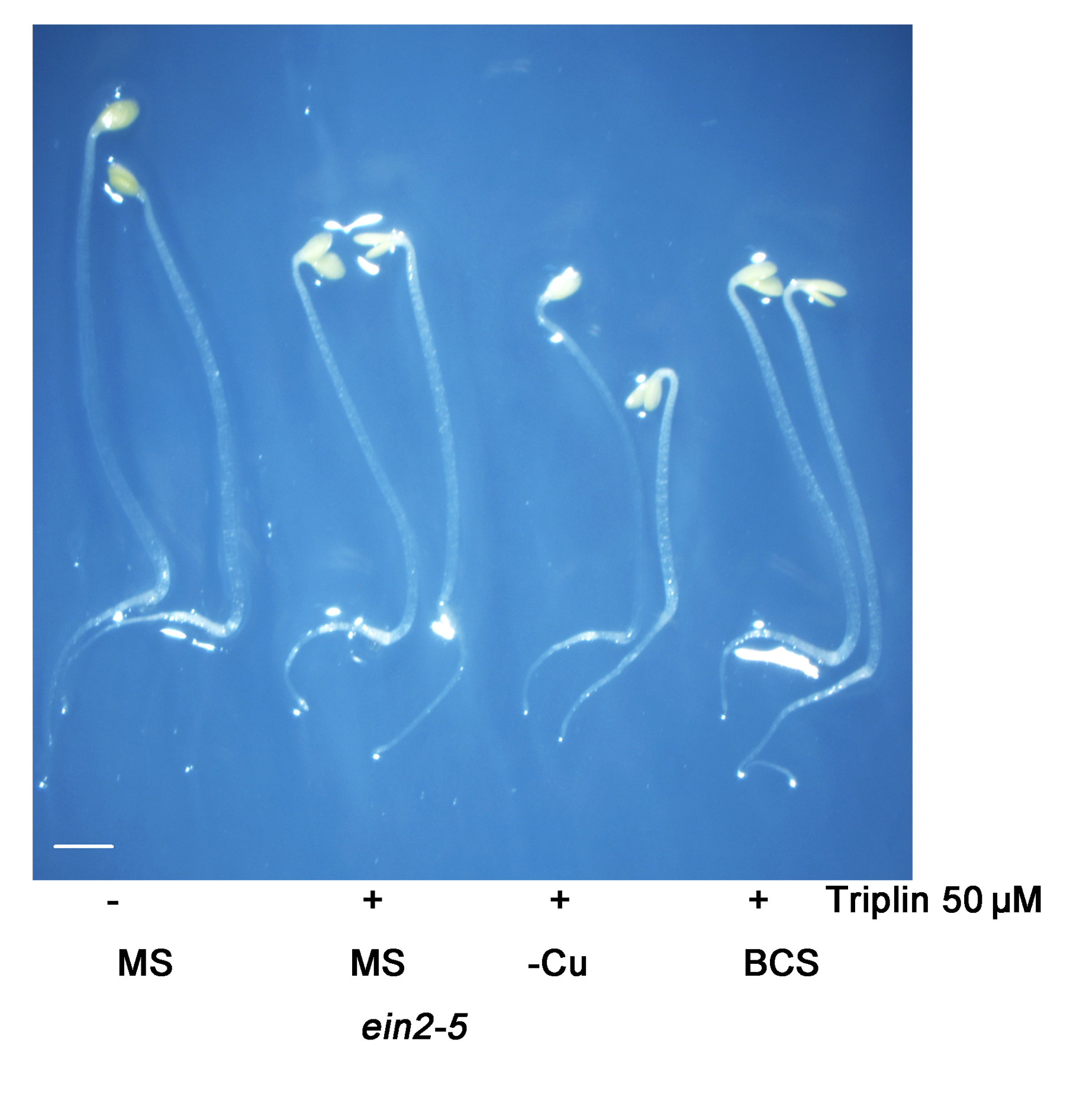

Supplement: S9 Fig — The phenotypes of 3-day-old, dark-grown ein2-5 seedlings grown on different growth medium with or without 50 μM triplin. -Cu indicates the growth medium was made of plant essential elements except copper ion. BCS indicates the growth medium of 0.5xMS with 500 μM BCS. The scale bar represents 1 mm. (TIF) [file pgen.1006703.s009.tif]

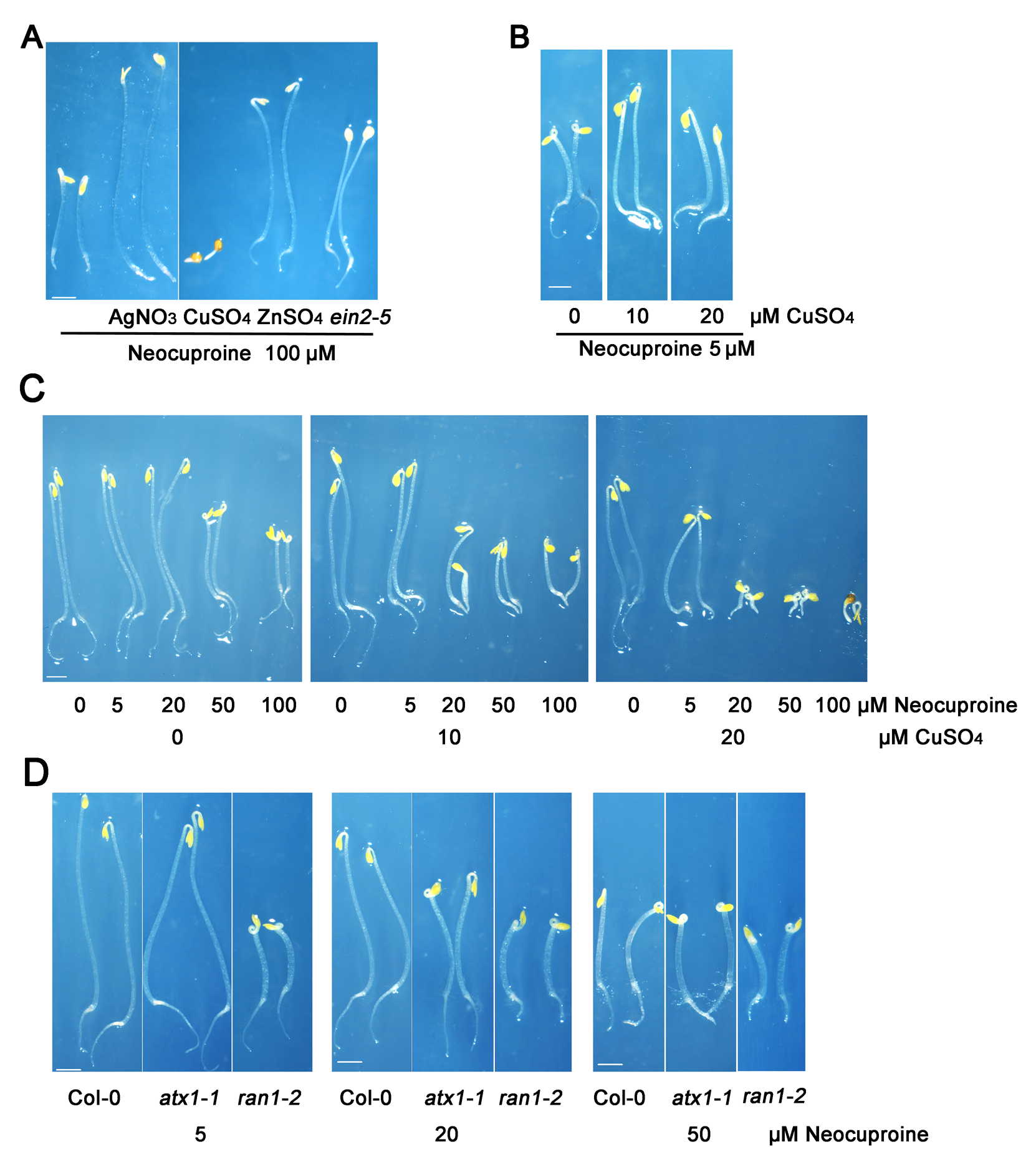

Supplement: S10 Fig — (A) The phenotypes of 3-day-old, dark-grown Col-0 seedlings treated with 100 μM neocuproine in the presence of no metal, 500 μM AgNO3, 100 μM CuSO4 or 100 μM ZnSO4. For comparison, the phenotypes of ein2-5 grown with only 100 μM neocuproine are shown. (B) The phenotypes of 3-day-old, dark-grown ran1-2 seedlings grown on 5 μM neocuproine without or with 10 or 20 μM CuSO4 are shown. (C) The phenotypes of 3-day-old, dark-grown Col-0 seedlings without or with 5, 20, 50 or 100μM neocuproine coupled with 0, 10 or 20 μM CuSO4 are shown. (D) atx1-1 and ran1-2 are hypersensitive to neocuproine. The phenotypes of 3-day-old, dark-grown seedlings of Col-0, atx1-1 and ran1-2 were treated with 5, 10 or 20 μM neocuproine. In all pictures, the scale bars represent 1 mm. (TIF) [file pgen.1006703.s010.tif]

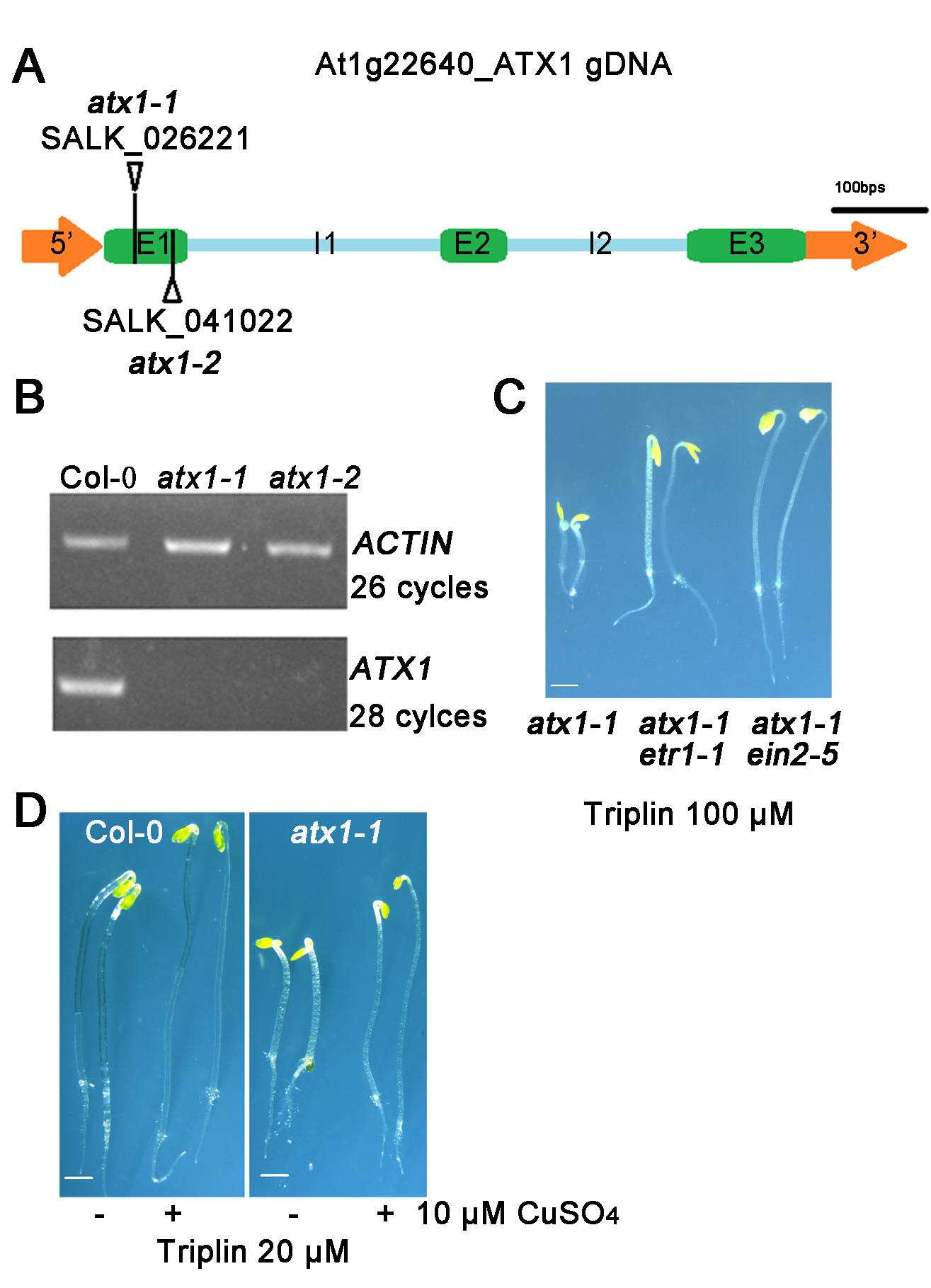

Supplement: S11 Fig — (A) A diagram shows the locations of the ATX1 T-DNA insertion mutants, Salk_026221 (atx1-1) and Salk_041022 (atx1-2). (B) The gene expression of the atx1-1 and atx1-2 were examined by RT-PCR. ACTIN was used as a loading control. (C) The phenotypes of 3-day-old, dark-grown seedlings of atx1-1, atx1-1 etr1-1 and atx1-1 ein2-5 treated with 100 μM triplin. The scale bar represents 1 mm. (D) The phenotypes of 3-day-old, dark-grown seedlings of Col-0 and atx1-1 treated with 20 μM triplin with (+) or without (-) 10μM CuSO4. The scale bars represent 1 mm. (TIF) [file pgen.1006703.s011.tif]

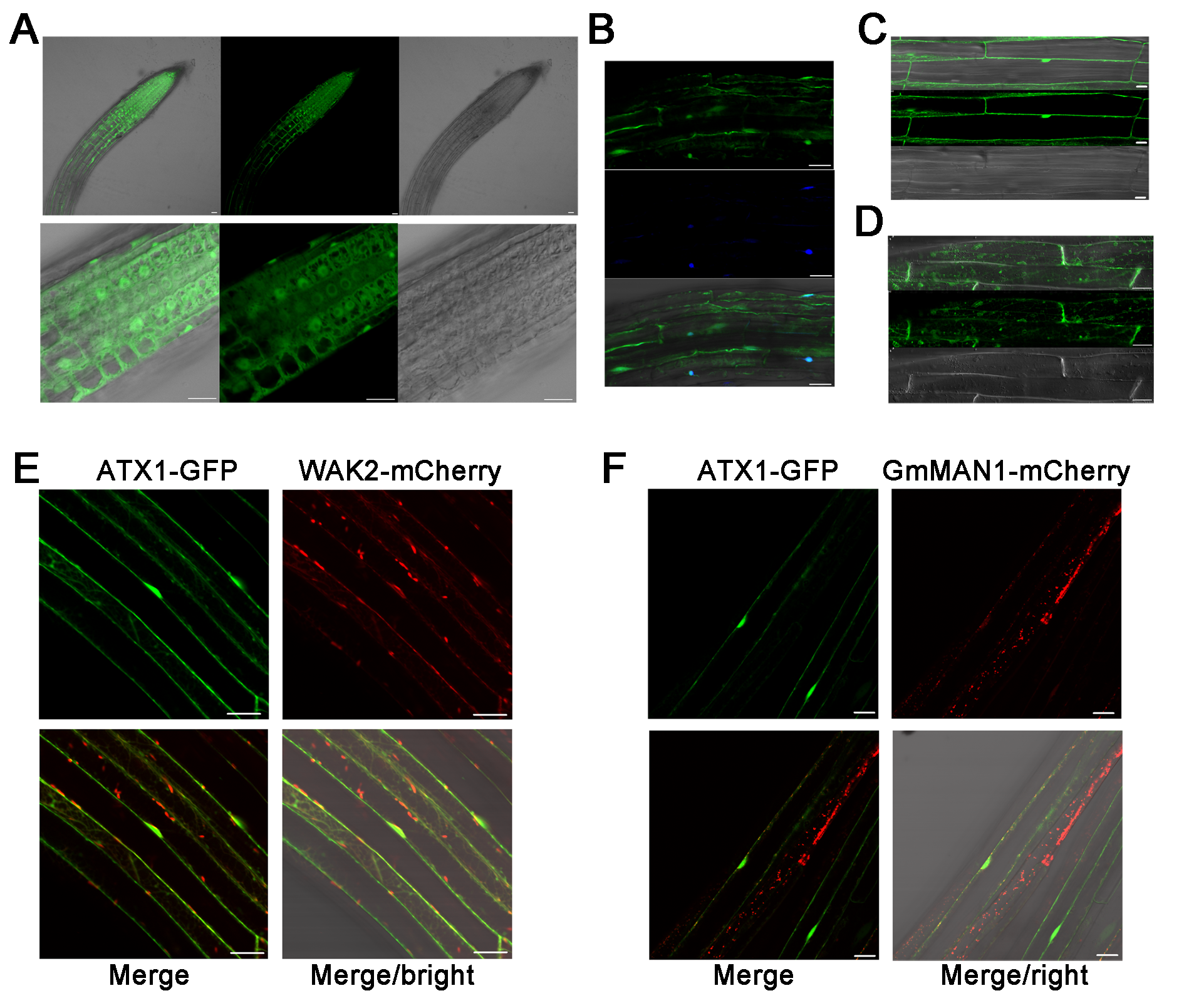

Supplement: S12 Fig — (A) Scanning images of the roots of 35S:ATX1-GFP (atx1-1) seedlings under a confocal microscope (Olympus FV1000). (B) Scanning images of the roots of 35S:ATX1-GFP (atx1-1) seedlings after DAPI staining under a confocal microscope (Olympus FV1000). (C) Scanning images of the epidermal cells of the hypocotyl of 35S:ATX1-GFP (atx1-1) seedling by a confocal microscope (NIKON A1R). (D) Scanning image of the bottom of a cell in the hypocotyl of 35S:ATX1-GFP (atx1-1) seedling by a confocal microscope (NIKON A1R). (E) Scanning images of the hypocotyls of F1 from 35S:ATX1-GFP (atx1-1) and transgenic marker lines with WAK2-mCherry under a confocal microscope (Olympus FV1000). (F) Scanning images of the hypocotyls of F1 from 35S:ATX1-GFP (atx1-1) and transgenic marker lines with GmMAN1-mCherry under a confocal microscope (Olympus FV1000). All scale bars represent 20 μm. (TIF) [file pgen.1006703.s012.tif]

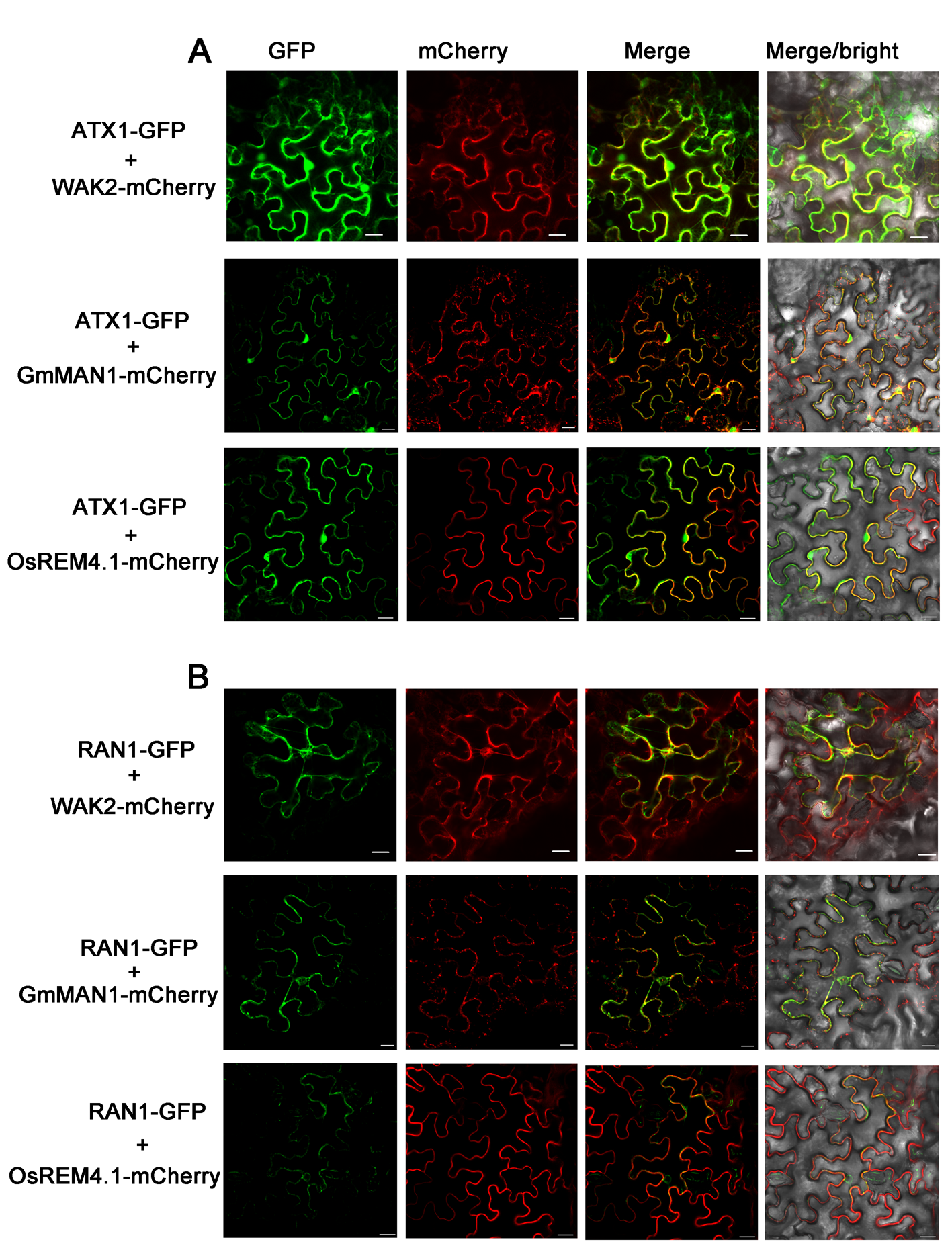

Supplement: S13 Fig — (A) ATX1-GFP and WAK2 / GmMAN1 / OsREM4.1-mCherry were transiently expressed in N. benthamiana leaves and their co-localizations were shown. (B) RAN1-GFP and WAK2 / GmMAN1 / OsREM4.1-mCherry were transiently expressed in N. benthamiana leaves and their co-localizations were shown. All images were made under a confocal microscope (Olympus FV1000).Scale bars represent 20 μM. (TIF) [file pgen.1006703.s013.tif]

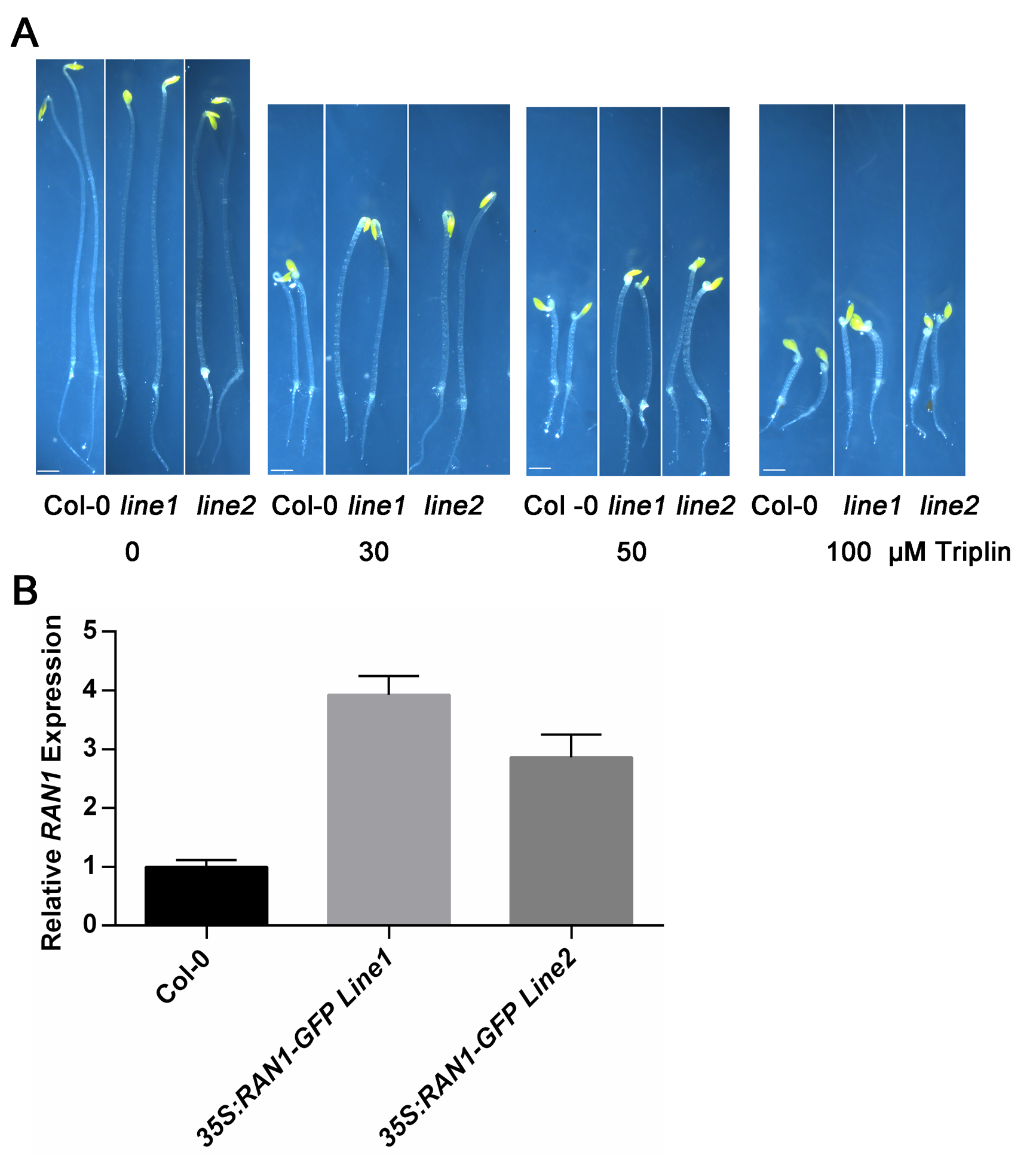

Supplement: S14 Fig — (A) The phenotypes of 3-day-old, dark-grown seedlings of Col-0 and two 35S:RAN1-GFP transgenic lines treated with 0, 30, 50 or 100 μM triplin. (B) qRT-PCR analysis of the relative RAN1 expression levels in two 35S:RAN1-GFP lines. Each experiment was repeated three times, and error bars represent SEM. (TIF) [file pgen.1006703.s014.tif]
