# Supplementary material for: Triplin, a small molecule, reveals copper ion transport in ethylene signaling from ATX1 to RAN1
Source: PLoS Genet. 2017 Apr 7;13(4):e1006703. doi: 10.1371/journal.pgen.1006703 (PMC5400275; doi:10.1371/journal.pgen.1006703)
Supplement: S1 Table — (DOCX) [file pgen.1006703.s015.docx]

**Supplemental table**

**Table S1. Structure of triplin like chemicals**

| Structure | PubChem CID | Phenotype? | Concentration |
| --- | --- | --- | --- |
| 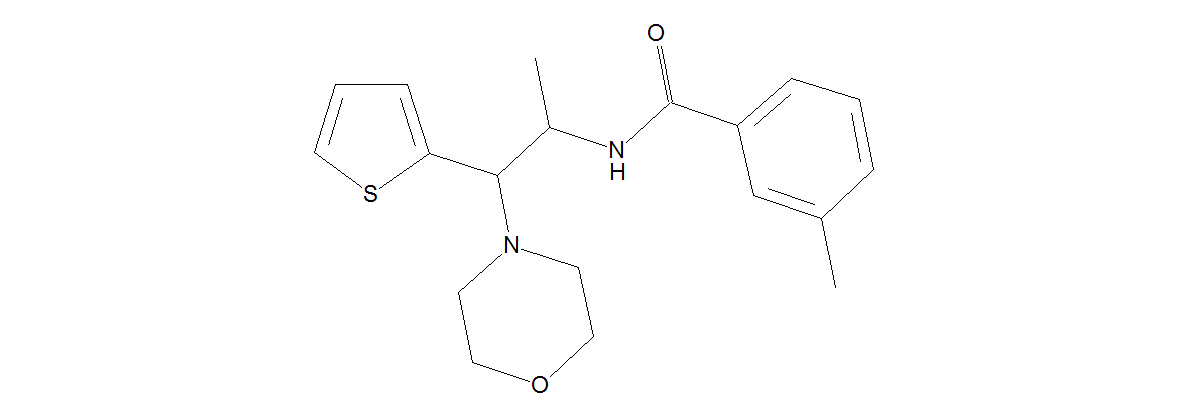 | F0655-0047 | Yes | 200 μM |
| 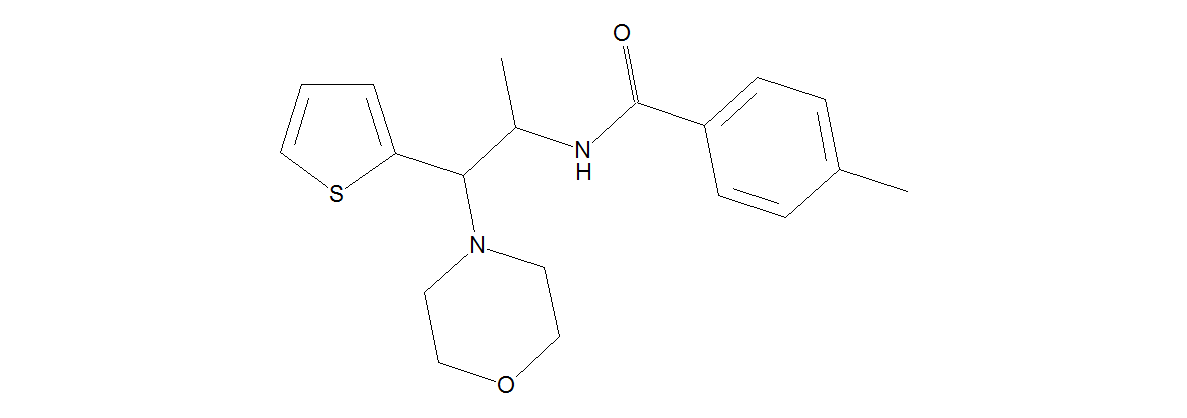 | F0655-0048 | Yes | 200 μM |
| 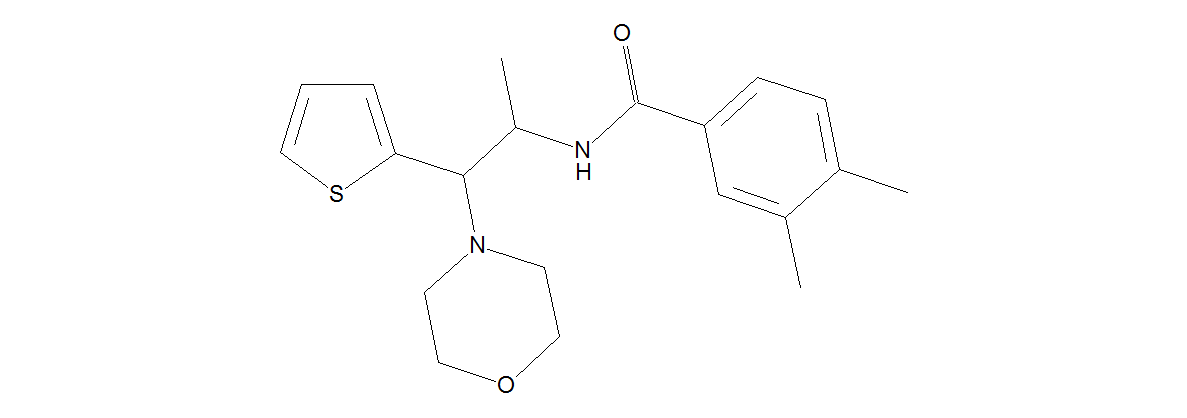 | F0655-0050 | Yes | 100 μM |
| 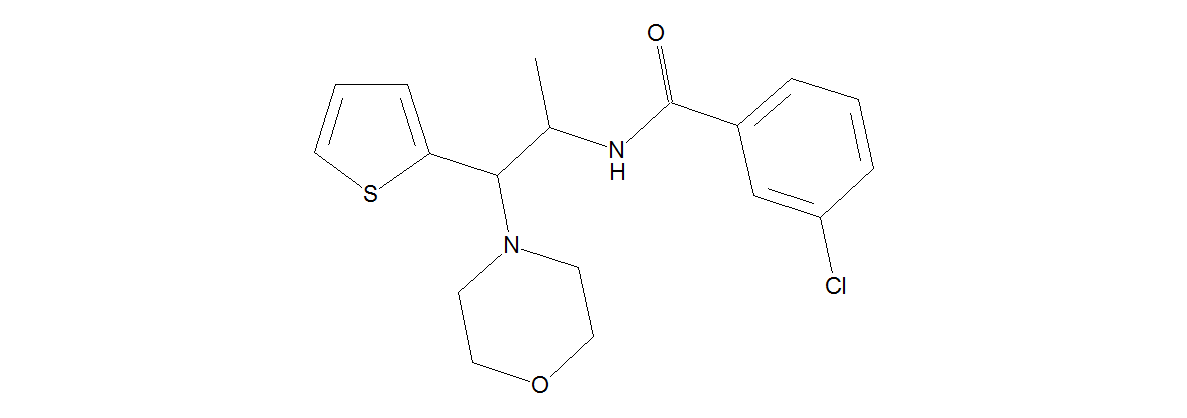 | F0655-0067 | Yes | 100 μM |
| 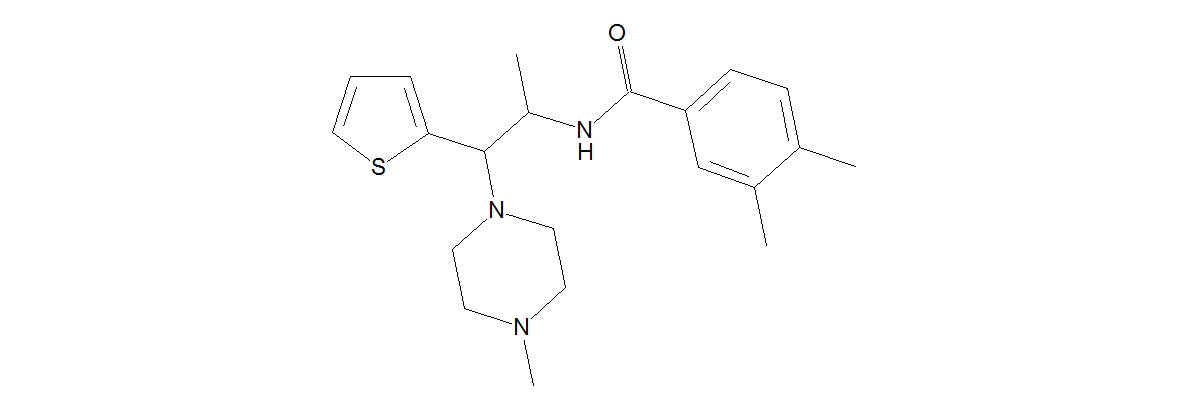 | F0655-0135 | No | 200 μM |
| 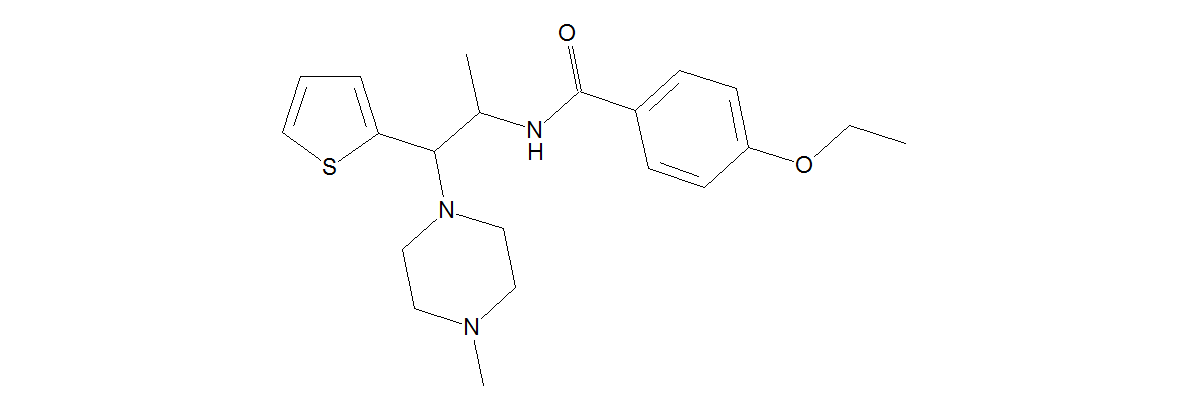 | F0655-0141 | No | 200 μM |
| 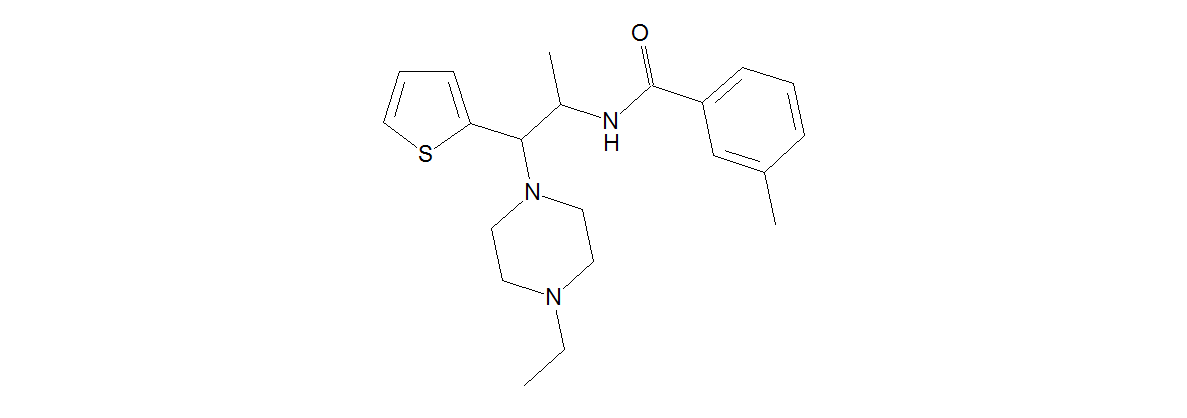 | F0655-0217 | No | 200 μM |
| 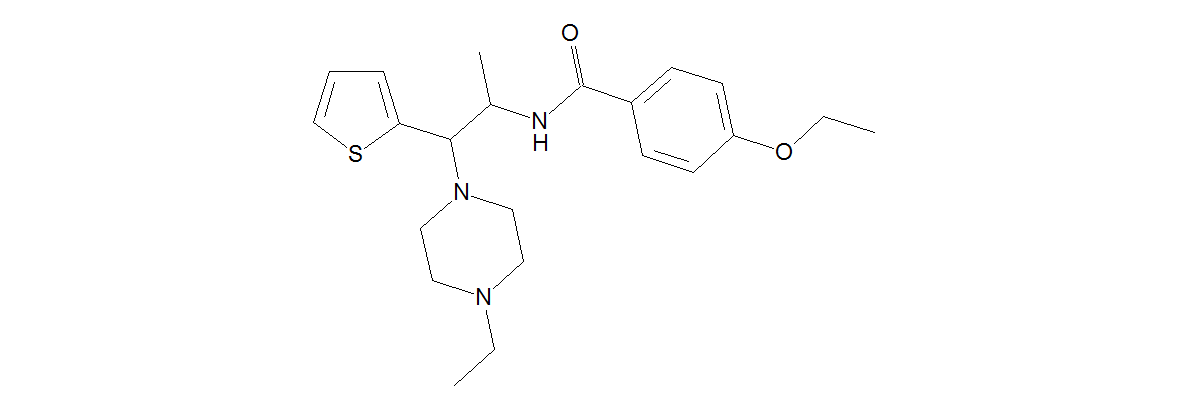 | F0655-0226 | No | 200 μM |
| 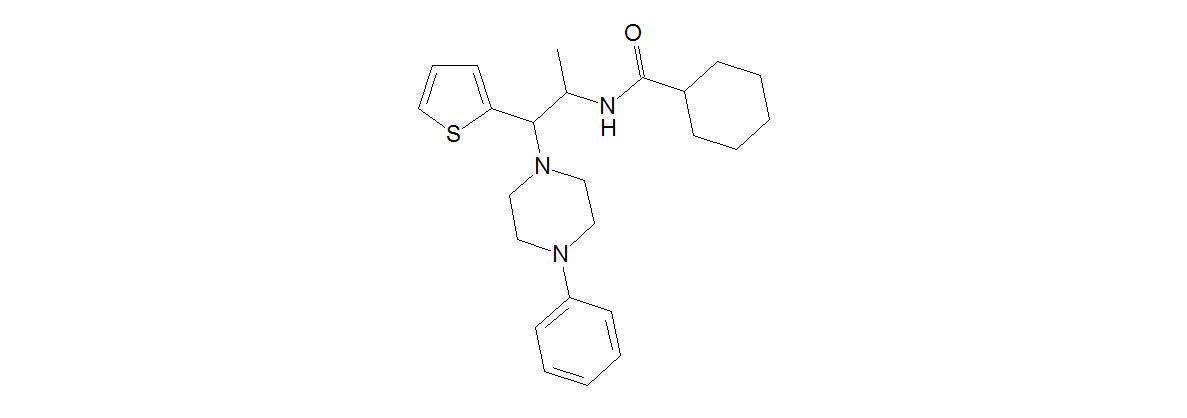 | F0655-0299 | No | 200 μM |
| 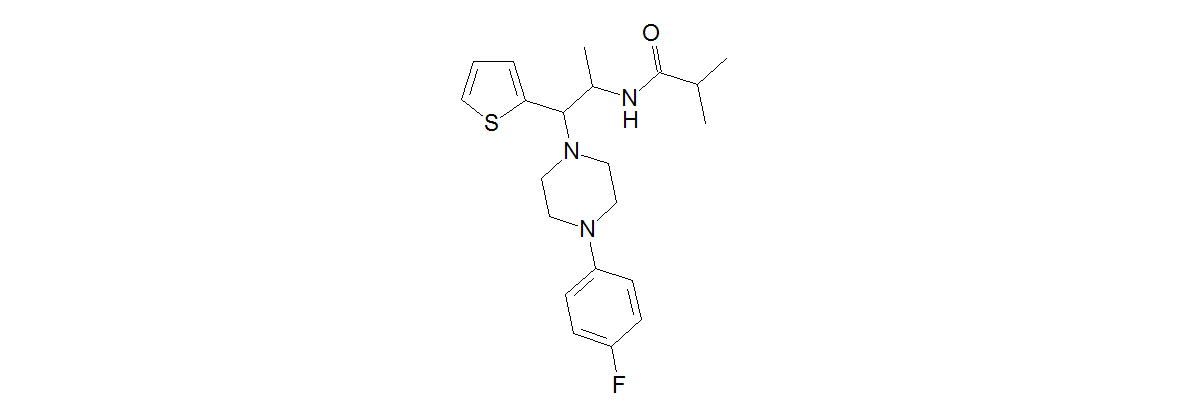 | F0655-0550 | No | 200 μM |
| 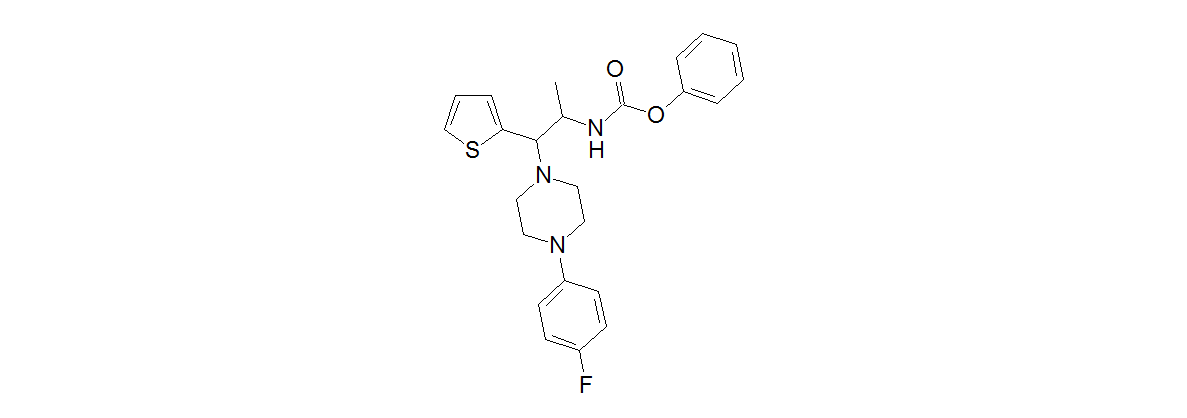 | F0655-0595 | Yes | 200 μM |
| 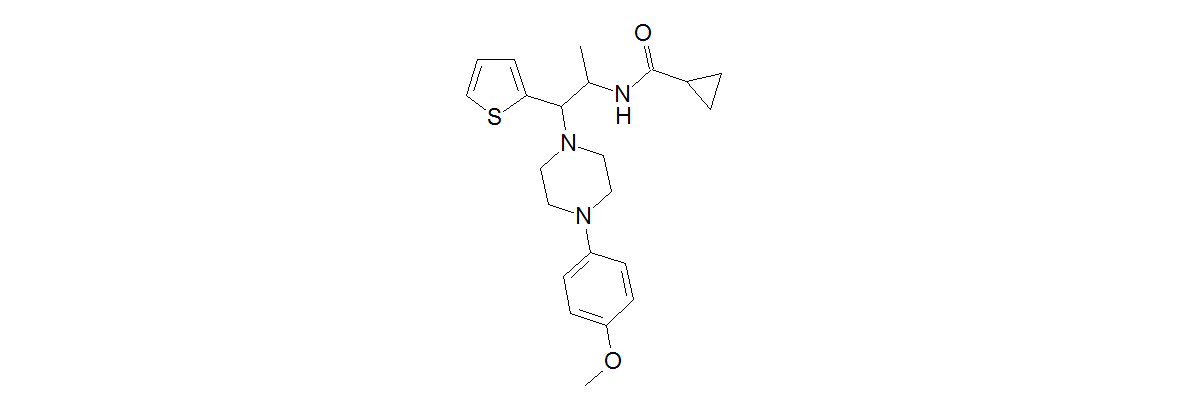 | F0655-0638 | No | 200 μM |
| 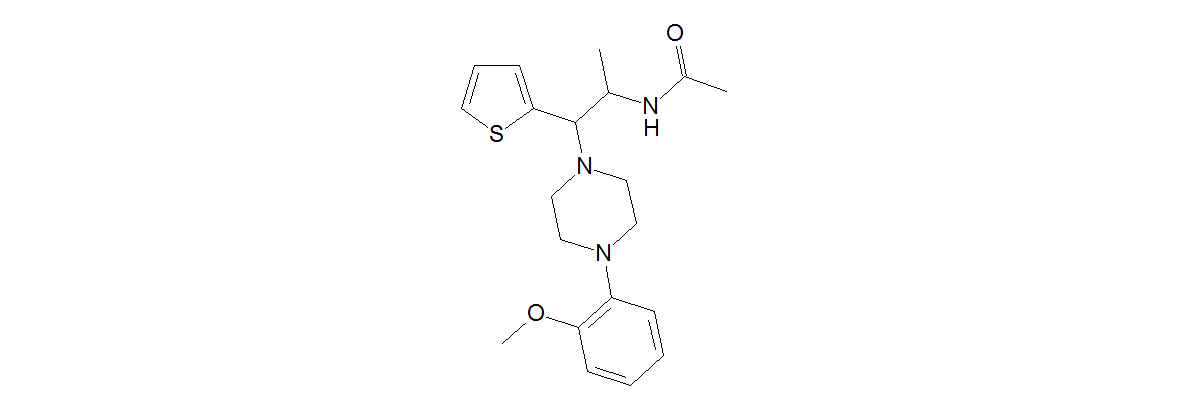 | F0655-0717 | No | 200 μM |
| 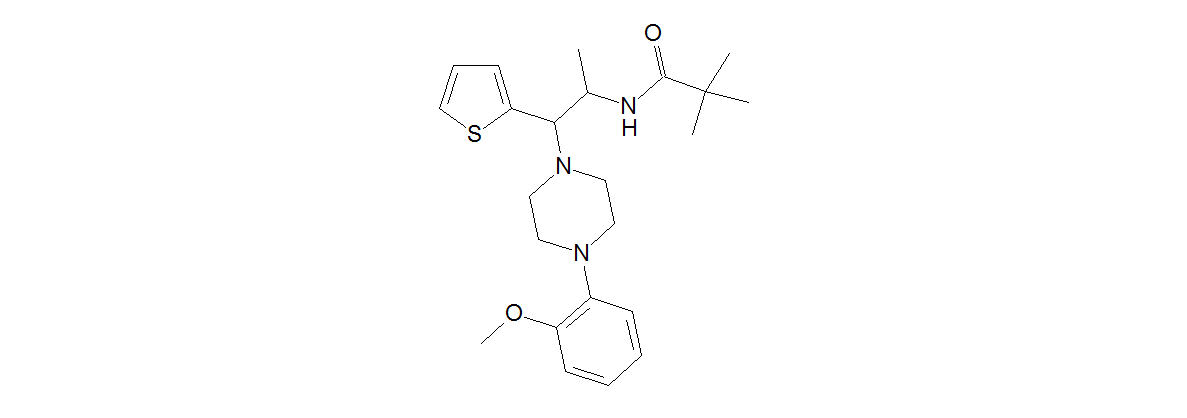 | F0655-0721 | Yes | 200 μM |
| 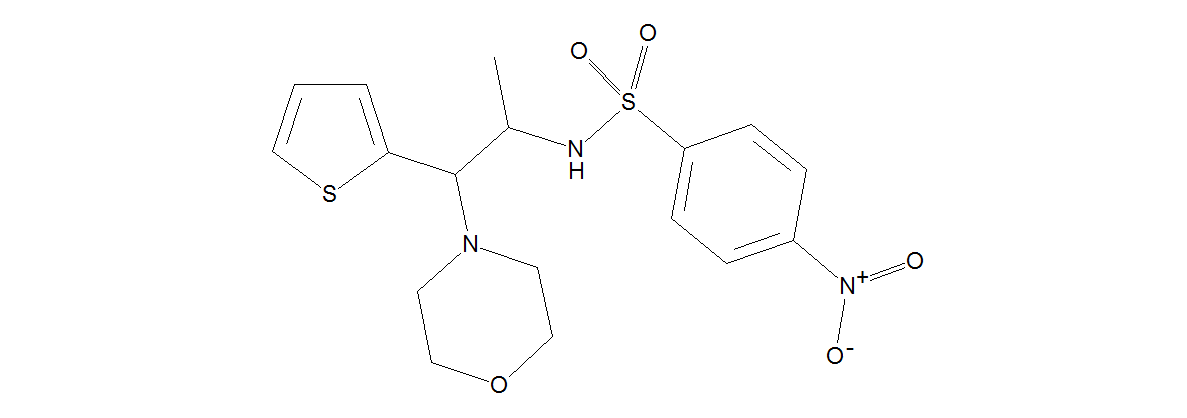 | F0655-0773 | no hook ,no root | 100 μM |
| 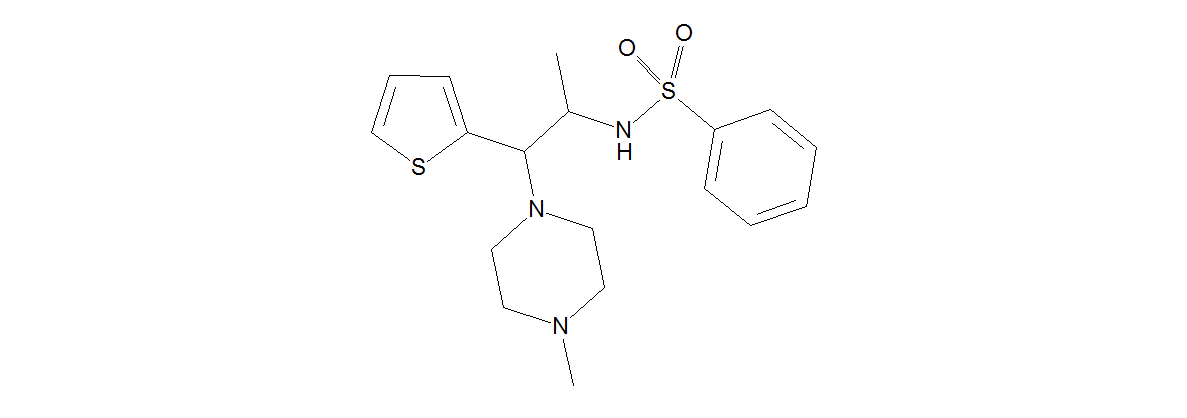 | F0655-0776 | No | 200 μM |
| 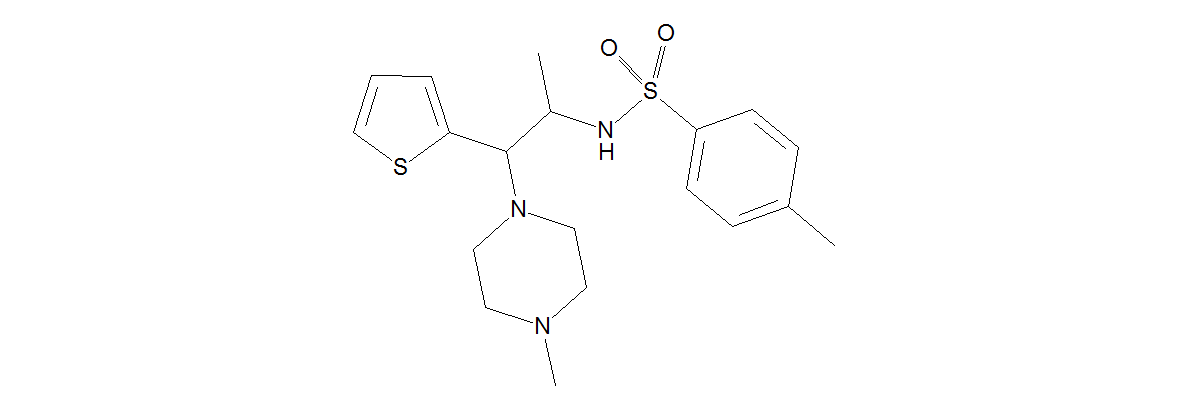 | F0655-0777 | Yes | 200 μM |
| 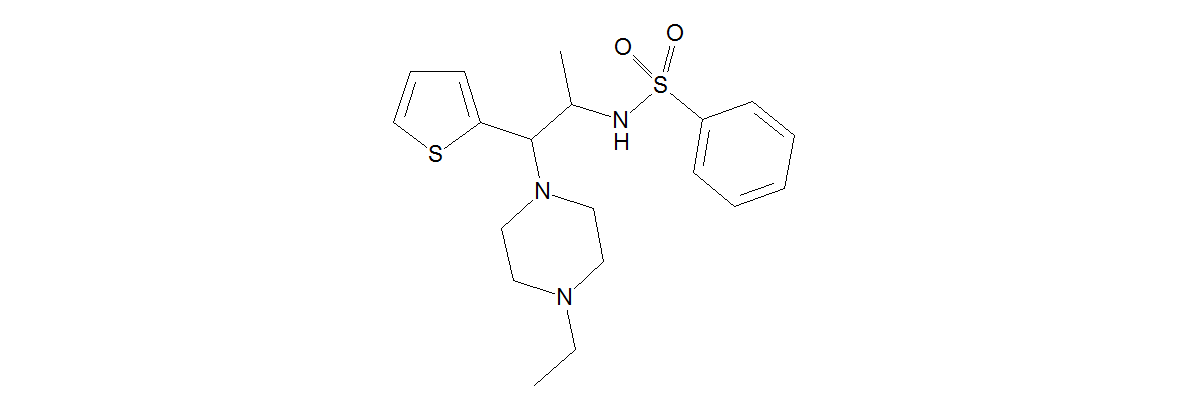 | F0655-0784 | No | 200 μM |
| 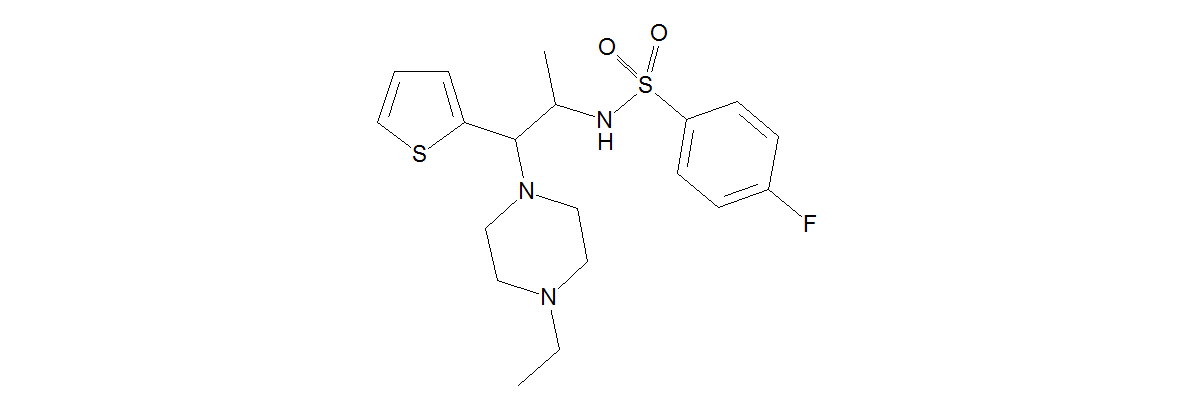 | F0655-0788 | No | 200 μM |
| 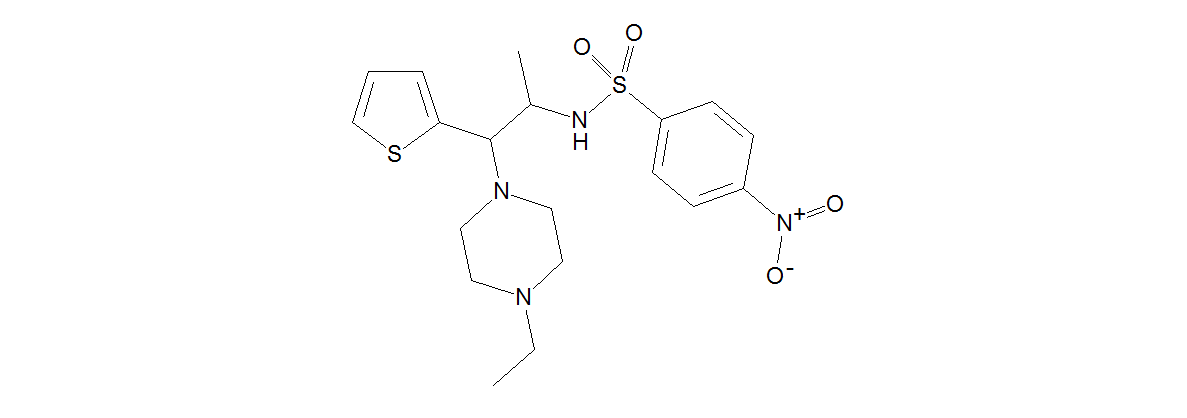 | F0655-0789 | No | 200 μM |
| 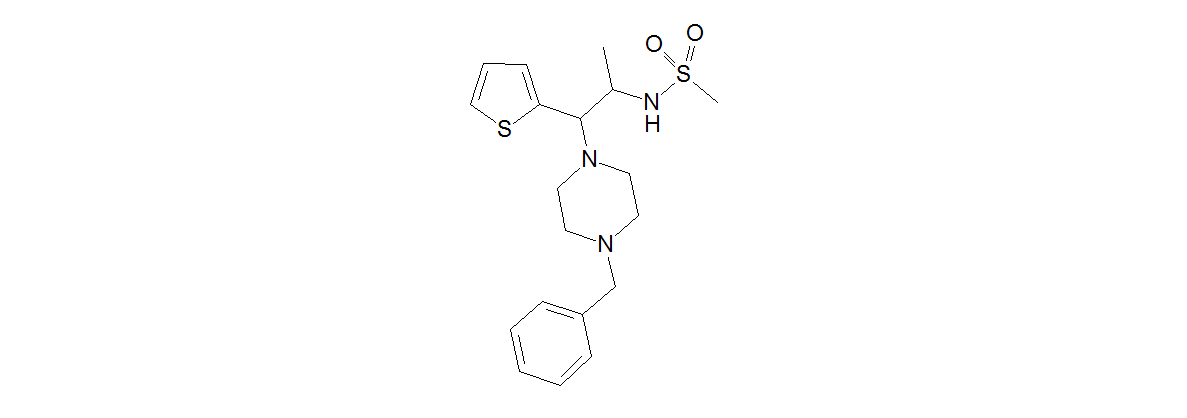 | F0655-0798 | No | 200 μM |
| 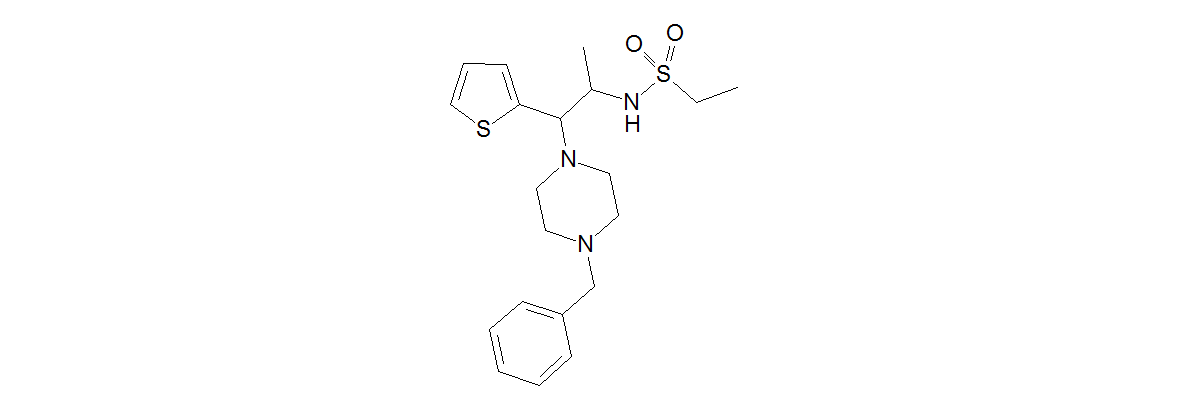 | F0655-0799 | No | 200 μM |
| 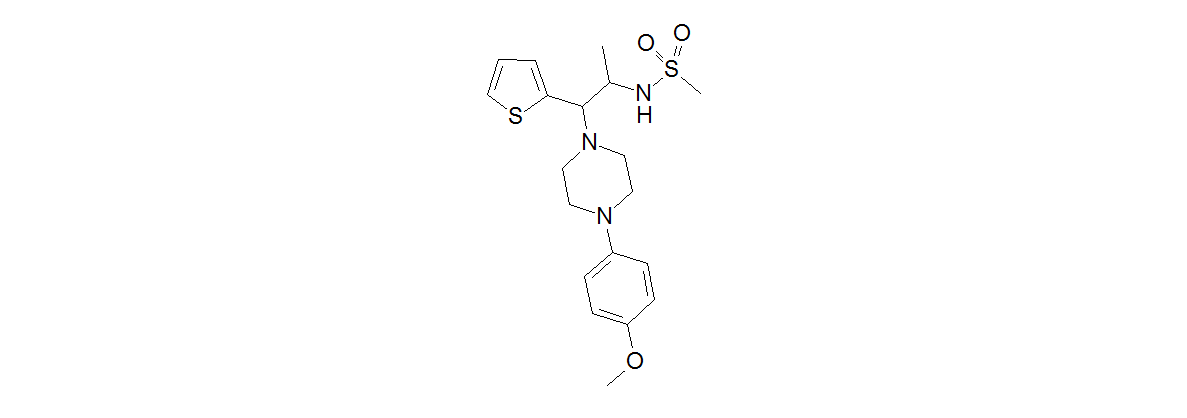 | F0655-0822 | Yes | 200 μM |
| 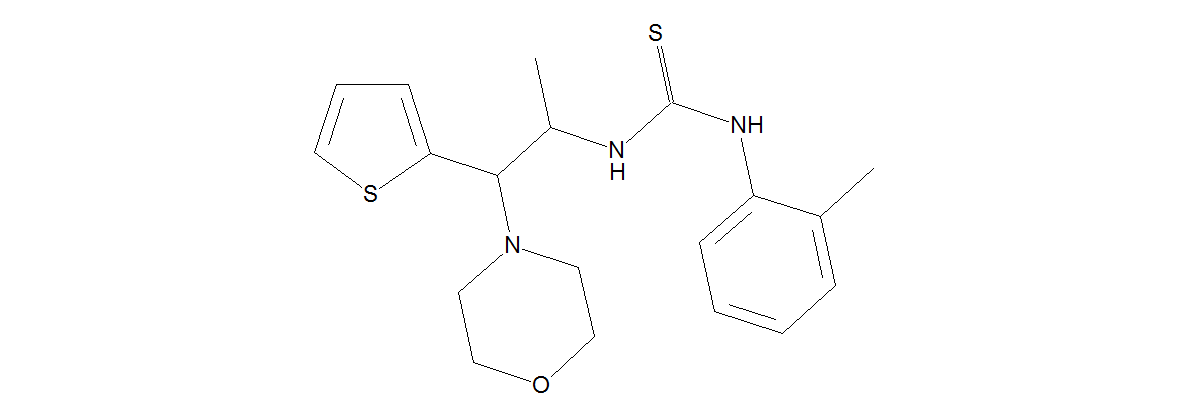 | F0655-0845 | Yes | 200 μM |
| 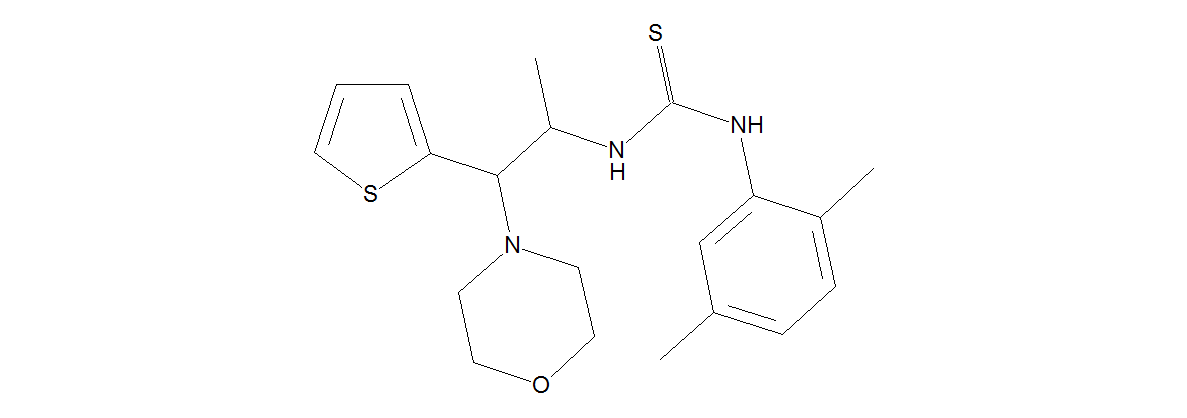 | F0655-0849 | Yes | 200 μM |
| 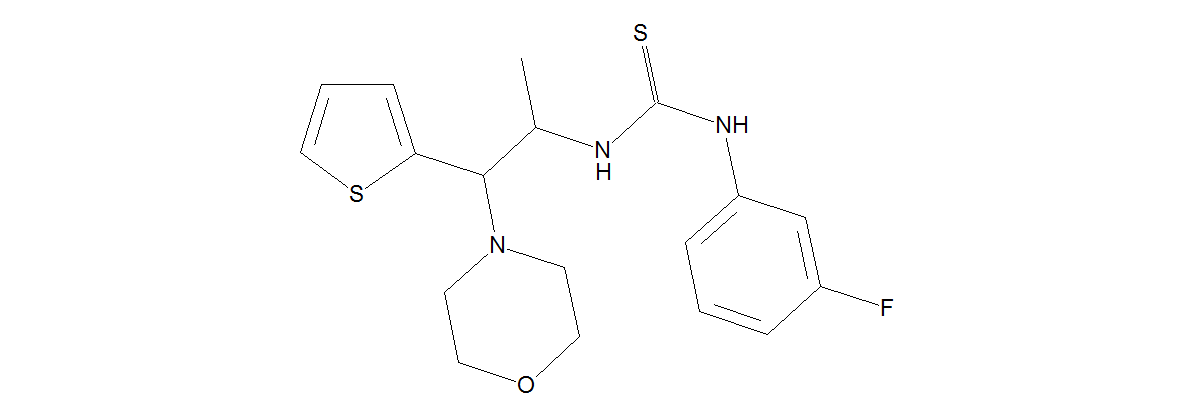 | F0655-0859 | Yes | 100 μM |
| 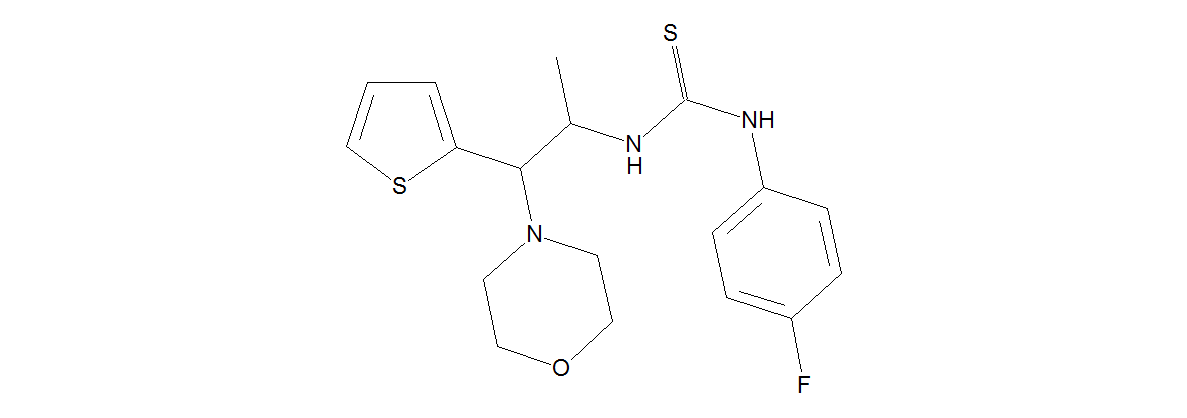 | F0655-0860 | Yes | 100 μM |
| 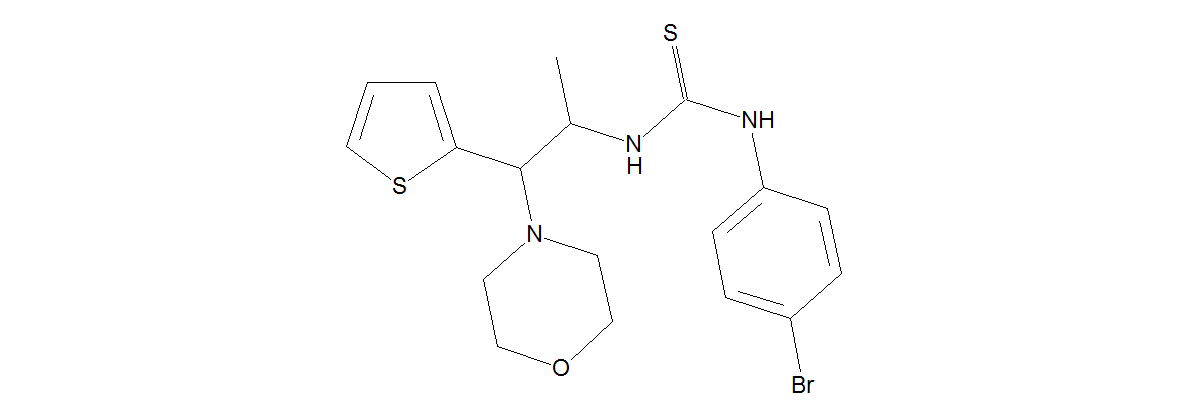 | F0655-0866 | Yes | 100 μM |
| 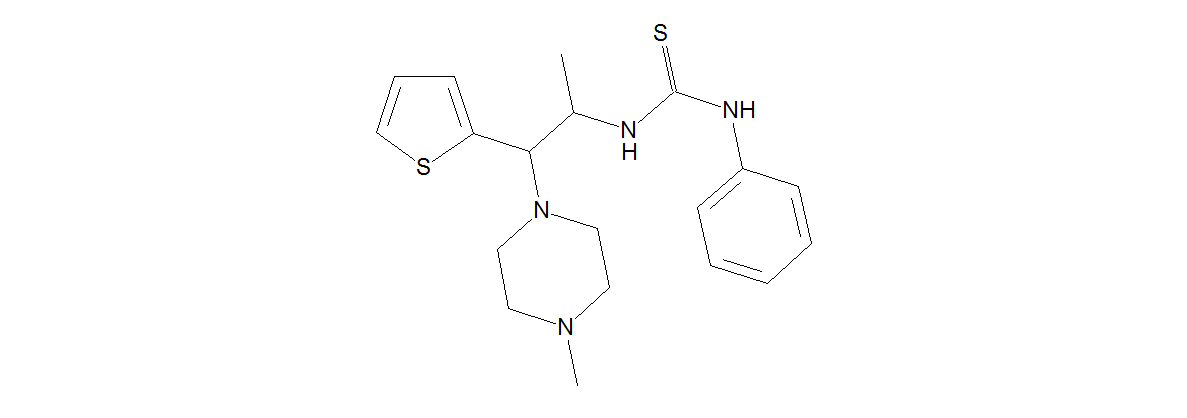 | F0655-0876 | No | 200 μM |
| 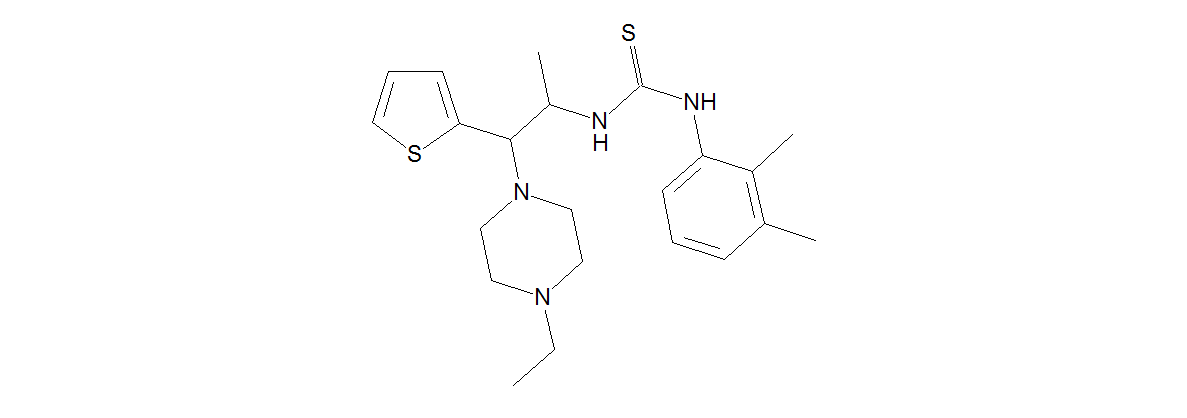 | F0655-0912 | No | 200 μM |
| 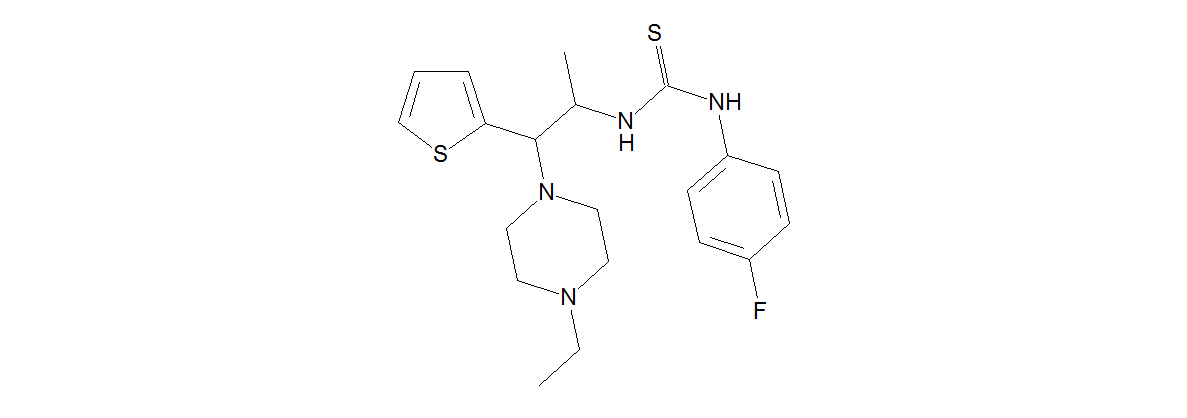 | F0655-0924 | No | 200 μM |
| 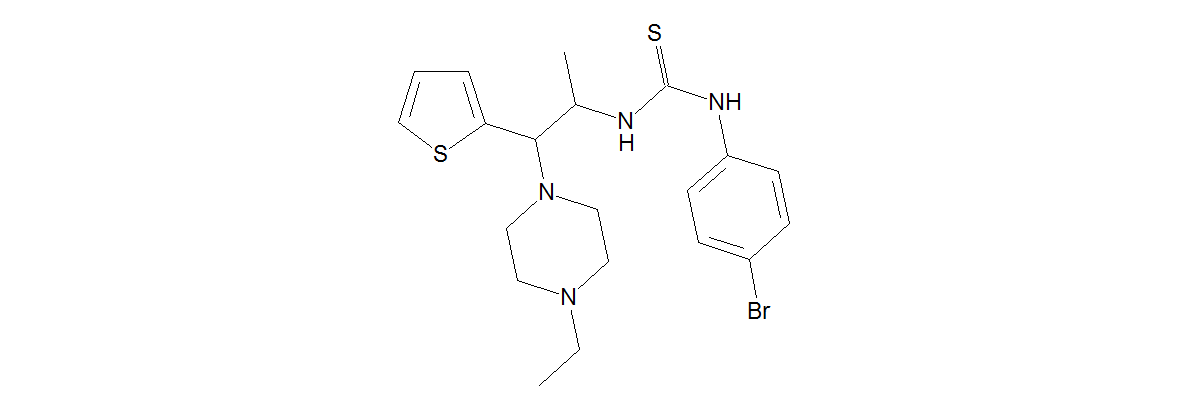 | F0655-0930 | Yes | 200 μM |
| 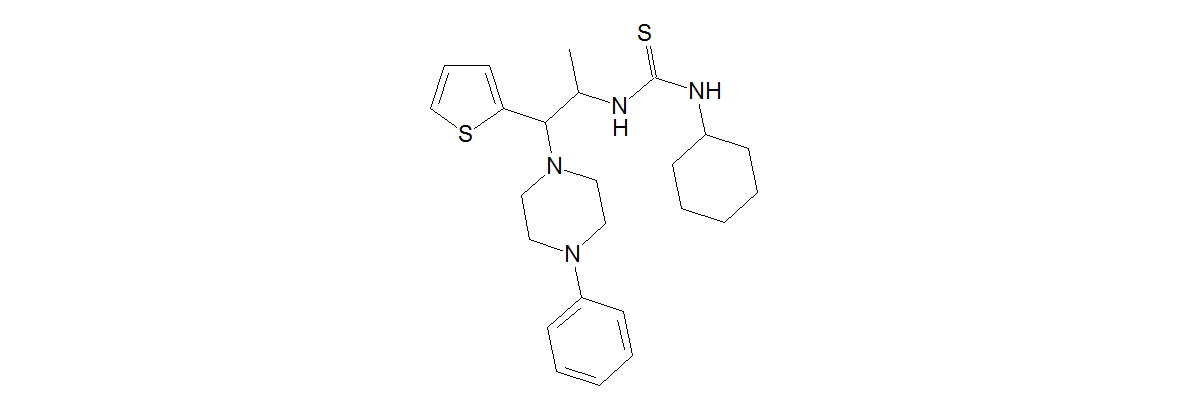 | F0655-0938 | Yes | 200 μM |
| 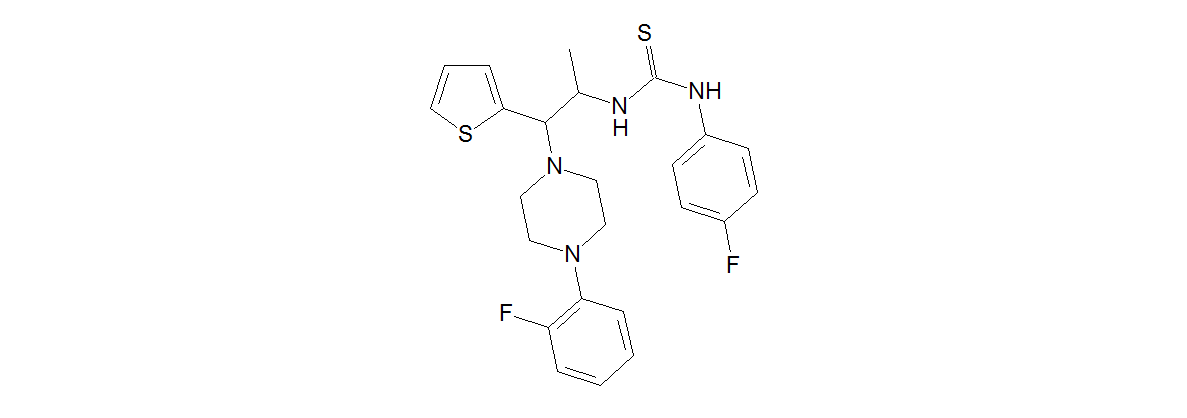 | F0655-1020 | No | 200 μM |
| 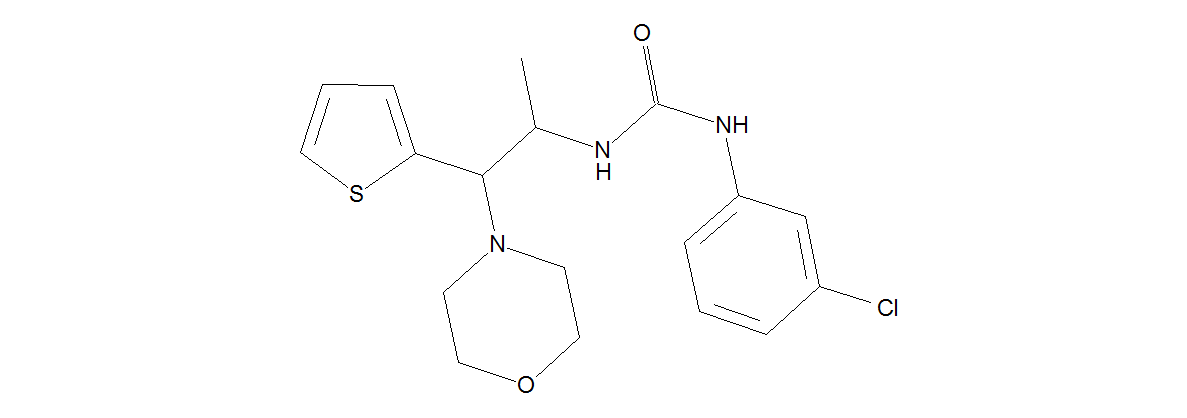 | F0655-1128 | no hook ,no root | 100 μM |
| 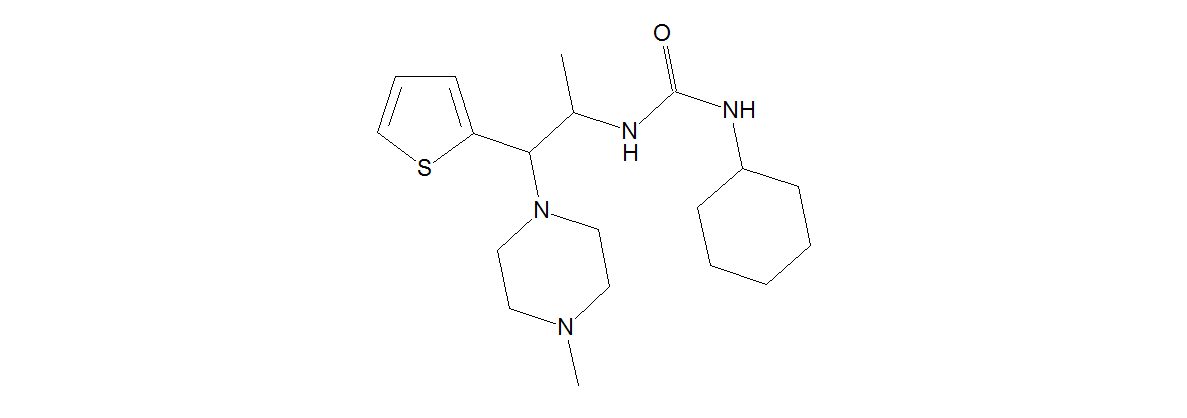 | F0655-1132 | No | 200 μM |
| 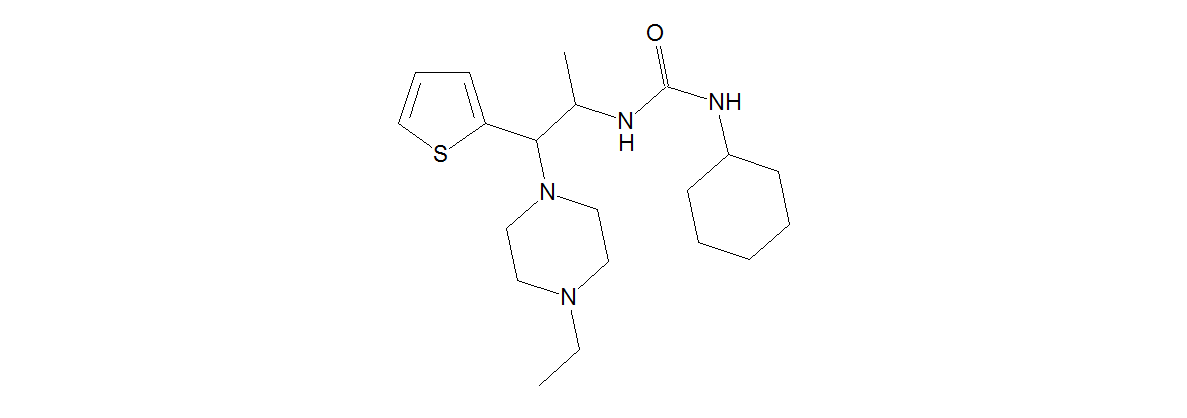 | F0655-1137 | No | 200 μM |
| 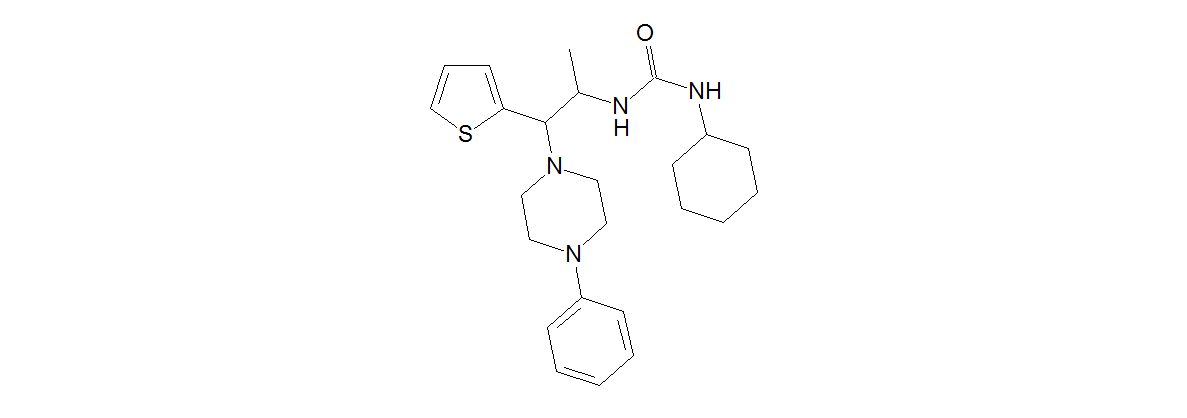 | F0655-1142 | Yes | 200 μM |
