# Supplementary material for: Triplin, a small molecule, reveals copper ion transport in ethylene signaling from ATX1 to RAN1
Source: PLoS Genet. 2017 Apr 7;13(4):e1006703. doi: 10.1371/journal.pgen.1006703 (PMC5400275; doi:10.1371/journal.pgen.1006703)
Supplement: S2 Table — (DOCX) [file pgen.1006703.s016.docx]

**Supplemental table**

**Table S2.** **Primers used in this research**

| Primer Name | DNA Sequence (5’-3’) |  |
| --- | --- | --- |
| ERF1-F | CGGAGAGAGTTCAAGAGTCGC | |
| ERF1-R | CTCCGTCTCATCGAGTGTTTCC | |
| ACTIN-Q-F | CGTTTCGCTTTCCTTAGTGTTA | |
| ACTIN-Q-R | AGCGAACGGATCTAGAGACTC | |
| ATX1-F-attb | GGGGACAAGTTTGTACAAAAAAGCAGGCTCCATGCTTAAAGACTTGTTCCAAGC | |
| ATX1-R-attb | GGGGACCACTTTGTACAAGAAAGCTGGGTTAGCCTTAGCAGTTTCACCTT | |
| ATX1-Q-F | TTTCAAGCCTTGTCGGTGGT | |
| ATX1-Q-R | GCTCCAACACATCCCTCACA | |
| ATX1-221-LP | TGCCACTTATACTCTCAAACGG | |
| ATX1-221-RP | CATGGCCACTCTGAGAACAAC | |
| ATX1-022-LP | GTTTTCTTTCCGGTTTTCGTC | |
| ATX1-022-RP | TTTTAGGACATTTCTGAGAATTCC | |
| RAN1-Q-F | ACCGCCCAAATGTCAAAAGC | |
| RAN1-Q-R | CTCCACCAATTGACCAGCCT | |
| RAN1F-attb | GGGGACAAGTTTGTACAAAAAAGCAGGCTCCATGGCGCCGAGTAGACGGGATT | |
| RAN1R-attb | GGGGACCACTTTGTACAAGAAAGCTGGGTTCTCCGTGGTGATTTTCAAAAC | |
| RAN1-YTH-R-attb | GGGGACCACTTTGTACAAGAAAGCTGGGTTAGTATCTTTCGAAGACAACCTTTCA | |
| ACTIN-RT-F | ATGCCCCAGGACATCGTGATTTCAT | |
| ACTIN-RT-R | TTGGCGGCACCCTTACGTGGATCA | |
| ACS5-Q-F | ACGCGGGTTTGTTCTGTTGGGT | |
| ACS5-Q-R | CCAACCCGGTTCGGTGCAGT | |
| ACS6-Q-F | GGAGGAGACTAAACCGATGGCTGC | |
| ACS6-Q-R | GGCACAGGCGAATGAGGCGA | |
| ACS11-Q-F | CCAGGCTCATCGTGTCATTGCGA | |
| ACS11-Q-R | GCAACCTCCATCGTTTGGTCCGA | |
